# Team performance evaluation in the 2025 FIBA Men’s Asia Cup: an entropy-weighted rank-sum ratio analysis with a case study of the China–Australia final

**DOI:** 10.3389/fspor.2026.1919832

**Published:** 2026-09-15

**Authors:** Zehui Zhang, Li Chen, Mingang Guo, Qilin Hu

**Affiliations:** School of Physical Education, Wuhan University of Technology, Wuhan, Hubei, China

**Keywords:** basketball technical statistics, comprehensive evaluation, entropy weight method, FIBA Men's Asia Cup, weighted rank-sum ratio

## Abstract

The evaluation of basketball performance has become increasingly multidimensional. However, relatively few studies have connected tournament-wide performance profiles with performance deviations in key matches, limiting the simultaneous interpretation of teams' relative competitive status and final-match statistical differences. Using official FIBA Box Score data from all 36 games of the 2025 FIBA Men's Asia Cup, a team-game-level multidimensional dataset was constructed. An entropy-weighted rank-sum ratio (WRSR) model was used to evaluate the comprehensive technical performance of the 16 participating teams, followed by tournament-level comparison and final-match deviation analysis of China and Australia. Assists, steals, offensive rebounds, and free throw percentage accounted for a cumulative entropy weight of 46.72%. Australia ranked first (WRSR = 0.7714) and was classified as Excellent, whereas China ranked seventh and was classified as Good. China showed higher shooting efficiency and fewer turnovers, while Australia recorded higher values for points, rebounds, assists, steals, and blocks. Relative to their pre-final baselines, China's free throw percentage increased by 10.5%, whereas offensive rebounds, assists, and steals decreased by 23.1%, 8.6%, and 34.8%, respectively. Australia's turnovers decreased by 53.1%, while assists and blocks increased by 19.8% and 42.9%. The final-match differences were concentrated mainly in possession control, offensive organization, defensive pressure, and rim protection. This study presents a reproducible tournament-level framework that integrates multidimensional technical evaluation with key-match deviation analysis, providing quantitative support for post-match diagnosis and between-match preparation while remaining complementary to real-time and possession-level performance analysis.

## 1 Introduction

Performance evaluation has become a central component of contemporary basketball performance analysis. Team performance emerges from interacting dimensions that include shooting efficiency, rebounding control, ball movement, possession stability, defensive pressure, and rim protection; consequently, no single box-score variable adequately represents overall technical performance. Basketball research has therefore moved from isolated comparisons toward multivariable and model-based approaches. Official box scores remain particularly useful because they are standardized, accessible, and comparable across games, even though they do not capture tactical execution, spatial organization, or possession-level context (1). This combination of breadth and reproducibility makes them suitable for tournament-level descriptive evaluation when their limitations are explicitly recognized.

Basketball studies have linked game-related statistics to competitive performance in several settings. Mandić et al. (2) described long-term statistical differences between NBA and EuroLeague basketball; Mikołajec et al. (3) identified offensive and defensive variables associated with EuroLeague success; and Zhang et al. (4) used quantile regression to model the relationship between match outcome and performance in the 2019 FIBA Basketball World Cup. Research on critical moments has further shown that technical and tactical differences depend on execution within specific game contexts (5). These studies support a multidimensional view of performance, but they also reveal recurring analytical limitations. First, many investigations are organized around winner-loser discrimination or a restricted set of indicators rather than a tournament-wide composite profile. Second, analyses of one or a few key games may conflate persistent team characteristics with opponent-, stage-, or match-specific fluctuations. Third, composite evaluation is sensitive to how indicator redundancy, direction, scale, and weighting are handled. Rank-sum ratio approaches have been used to compare basketball team performance strategies (6), while entropy-based and network approaches have been applied to collective performance (7); however, their integration with a within-team baseline for interpreting a key match remains limited.

These methodological issues are not unique to basketball. Across sports, performance-analysis research has emphasized that useful indicators should be interpreted as multidimensional profiles rather than as isolated measures (8). Systematic reviews in football (9), invasion team sports (10), and rugby union (11) have documented the combined use of technical, tactical, physical, and contextual indicators, while an umbrella review of team-ball sports highlighted the importance of analytical level and match context when explaining team performance (12). At the same time, tracking-based frameworks increasingly combine multiple data streams to support performance monitoring (13), and technology-assisted feedback has long been used to translate observed performance into information for athletes and coaches (14). Collectively, this literature indicates that a robust evaluation framework should make indicator selection transparent, preserve comparability across observations, and convert multidimensional data into interpretable information without implying causality from descriptive metrics. The present study adopts these principles within the narrower setting of official basketball box-score data.

The FIBA Men's Asia Cup provides a suitable setting for this purpose because its official statistics are collected under a common competition structure and allow tournament-wide comparison among national teams. In the 2025 edition, China and Australia reached the final, creating a useful case for examining whether final-match statistical differences were consistent with, or departed from, each team's broader tournament profile. Interpreting the final in isolation would make it difficult to distinguish persistent tournament-level characteristics from short-term variation under a specific opponent and competition stage. Accordingly, the 16-team tournament dataset was used to establish relative technical profiles, while each finalist's pre-final performance was used as an internal baseline for assessing final-match deviation. An entropy weight method combined with a weighted rank-sum ratio (WRSR) model offers a transparent quantitative approach to this problem: entropy weighting assigns data-driven weights according to between-team dispersion, and WRSR converts multiple indicator ranks into a normalized team-level score. The resulting weights describe information discrimination within this sample rather than causal importance for winning, and the WRSR score represents relative comprehensive technical performance rather than a direct prediction of match outcome.

Accordingly, this study included all 36 games of the 2025 FIBA Men's Asia Cup and extracted team TOTAL rows from official FIBA Box Scores to construct 72 team-game records. Eleven technical indicators were retained: points, 2-point field goal percentage, 3-point field goal percentage, free throw percentage, offensive rebounds, defensive rebounds, assists, fouls, turnovers, steals, and blocks. The entropy-weighted WRSR model was used to evaluate the 16 participating teams, after which China and Australia were compared at the tournament level and their final-match deviations were quantified against pre-final baselines. Three research questions were addressed: (1) which technical indicators showed the greatest information discrimination among the 16 teams; (2) how the teams ranked in comprehensive technical performance and where China and Australia were positioned; and (3) how the two finalists deviated from their pre-final performance levels in the final and in which technical dimensions those deviations were concentrated. The aim was to develop a reproducible, official-statistics-based tournament evaluation framework that supports team assessment, structured post-match review, and preparation for subsequent key matches without positioning retrospective analysis as a substitute for real-time monitoring.

## 2 Materials and methods

### 2.1 Study design and data sources

This study adopted a retrospective observational design based on publicly available official game statistics. The sample included all 36 games of the 2025 FIBA Men's Asia Cup. A team-game record was the original unit of extraction, with the two teams in each game recorded separately, yielding 72 raw records. These records were subsequently aggregated by team to create tournament-level technical data for the 16 participating teams, which served as the evaluation objects in the entropy-weighted WRSR model.

Official FIBA Box Scores were the primary data source. For every team and game, data were extracted consistently from the team TOTAL row. When a Box Score contained a Team/Coaches statistical row, those values were already incorporated into the TOTAL row and were therefore not entered or calculated separately. Indicator selection was additionally informed by the basketball performance-analysis literature, but all model calculations, reported results, and conclusions were based on official FIBA Box Score data. No video-coded variables were included.

### 2.2 Indicator selection and data quality control

Indicator selection was completed before model construction using a prespecified two-stage procedure based on basketball-specific interpretability, mathematical relationships among box-score variables, and information overlap. An initial pool of 23 team-level technical indicators was assembled from variables consistently available in official FIBA Box Scores (Supplementary Table S1). In the first stage, variables that were direct mathematical components, derived measures, composite indices, or outcome measures were excluded when their simultaneous inclusion would duplicate information already represented by more elementary or interpretable indicators. In the second stage, Pearson correlation analysis of tournament-level team aggregates was used to identify additional information overlap among candidate variables. When highly correlated variables had closely related basketball interpretations, the variable that more directly represented technical efficiency or quality was preferentially retained. The complete screening decisions and correlation results are reported in Supplementary Tables S1–S3.

The resulting evaluation system comprised 11 indicators: points, 2-point field goal percentage, 3-point field goal percentage, free throw percentage, offensive rebounds, defensive rebounds, assists, fouls, turnovers, steals, and blocks (Table 1). Points, the three shooting-percentage indicators, offensive and defensive rebounds, assists, steals, and blocks were treated as positive indicators, whereas fouls and turnovers were treated as negative indicators. Shooting percentages were calculated from tournament-level totals of made and attempted shots rather than by averaging game-level percentages; all other count-type indicators were expressed as per-game values. This specification ensured that indicator direction and aggregation were fixed before standardization and weighting.

**Table 1:** Technical indicator system included in the entropy-weighted WRSR model.

Technical dimension	Indicator	Direction	Technical interpretation
Offensive output	Points	Positive	Reflects overall team offensive output
Shooting efficiency	2-point field goal percentage	Positive	Reflects mid-range and interior finishing efficiency
Shooting efficiency	3-point field goal percentage	Positive	Reflects perimeter shooting efficiency
Free throw efficiency	Free throw percentage	Positive	Reflects free throw stability
Rebounding control	Offensive rebounds	Positive	Reflects ability to create second-chance opportunities
Rebounding control	Defensive rebounds	Positive	Reflects defensive rebounding protection
Organization and teamwork	Assists	Positive	Reflects team coordination and offensive organization
Discipline control	Fouls	Negative	Reflects foul-control performance
Possession stability	Turnovers	Negative	Reflects possession control and offensive stability
Defensive pressure	Steals	Positive	Reflects defensive pressure and transition-opportunity creation
Rim protection	Blocks	Positive	Reflects interior defense and rim protection

Data quality control was performed at the team-game level before aggregation. Checks covered record completeness, Game ID consistency, team-opponent mirroring, score mirroring, score decomposition, and shooting-percentage consistency. Score decomposition was verified using 2FG made × 2 + 3FG made × 3 + FT made = team points. Only records passing all checks were eligible for model input. All 72 team-game records passed these checks and were retained; the raw records, check definitions, outcomes, and 16-team aggregated dataset are provided in Supplementary Table S4.

### 2.3 Construction of the entropy-weighted WRSR model

#### 2.3.1 Construction of the raw data matrix

Let *m* = 16 denote the evaluation objects (participating teams) and *n* = 11 denote the technical indicators. The tournament-level input matrix was defined as:

$$X=(x_{ij}{)}_{m\times n}$$ (1)

where *i* = 1, ..., *m*; *j* = 1, ..., *n*; and $x_{ij}$ is the value of indicator *j* for team *i*. For points, offensive rebounds, defensive rebounds, assists, fouls, turnovers, steals, and blocks, tournament-level per-game means were used. For 2-point field goal percentage, 3-point field goal percentage, and free throw percentage, total made shots were divided by total attempts across all games played by the team. This aggregation avoids unequal weighting of games when the number of attempts differs across matches.

#### 2.3.2 Directional standardization

Because the indicators differed in scale and evaluation direction, min-max range standardization was used to place all variables on a common 0–1 scale and align higher standardized values with more favorable performance. For positive indicators:

$$z_{ij}=\frac{x_{ij}-\underset{i}{\min}\;(x_{ij})}{\underset{i}{\max}\;(x_{ij})-\underset{i}{\min}\;(x_{ij})}$$ (2)

For negative indicators (fouls and turnovers):

$$z_{ij}=\frac{\underset{i}{\max}\;(x_{ij})-x_{ij}}{\underset{i}{\max}\;(x_{ij})-\underset{i}{\min}\;(x_{ij})}$$ (3)

The standardized matrix was:

$$Z=(z_{ij}{)}_{m\times n}$$ (4)

Accordingly, 0 ≤ *z_ij_* ≤ 1. No additive constant was applied to the standardized values. In the entropy calculation, when *p_ij_* = 0, the term *p_ij_* ln(*p_ij_*) was defined as 0 by continuity. This treatment preserves the observed dispersion and is consistent with the standardized and proportion matrices reported in Supplementary Table S5.

#### 2.3.3 Calculation of entropy weights

Entropy weighting was used to derive data-driven weights from between-team dispersion. For each indicator, the proportion of team *i* was calculated as:

$$p_{ij}=\frac{z_{ij}}{\sum_{i=1}^{m}z_{ij}}$$ (5)

The normalization constant was:

$$k=\frac{1}{\ln(m)}$$ (6)

and the information entropy of indicator *j* was:

$$e_{j}=-k\overset{m}{\underset{i=1}{\sum }}\;p_{ij}\ln(\;p_{ij})$$ (7)

where *k* ensures 0 ≤ *e_j_* ≤ 1. For *p_ij_* = 0, *p_ij_* ln(*p_ij_*) = 0. The information utility of each indicator was then calculated as:

$$d_{j}=1-e_{j}$$ (8)

and the entropy weight was:

$$w_{j}=\frac{d_{j}}{\sum_{\;j=1}^{n}\;d_{j}},\;\overset{n}{\underset{\;j=1}{\sum }}\;w_{j}=1$$ (9)

A larger weight indicates greater information dispersion across the 16 teams and therefore greater contribution to discrimination within this sample. It should not be interpreted as evidence that the indicator has a stronger causal effect on match outcome.

#### 2.3.4 Indicator ranking and WRSR calculation

After entropy weights were obtained, each team was ranked on each indicator. Ranks ranged from 1 to m. For positive indicators, larger raw values received higher ranks; for fouls and turnovers, smaller raw values received higher ranks. Tied values were assigned average ranks. Following established rank-sum ratio applications in sport and multivariate evaluation (15, 16), the weighted rank-sum ratio (WRSR) was calculated as:

$$\mathrm{WRS}R_{i}=\frac{1}{m}\overset{n}{\underset{\;j=1}{\sum }}\;w_{j}R_{ij}$$ (10)

where *R_ij_* is the rank of team *i* for indicator *j* and *w_j_* is the corresponding entropy weight. The denominator is *m*, the number of evaluation objects, because indicator ranks are defined on the 1-to-m scale. With *Σw_j_* = 1, division by m normalizes the weighted rank sum to a comparable scale. This notation explicitly distinguishes m (teams) from *n* (indicators). Complete indicator ranks and weighted rank contributions are provided in Supplementary Table S6, and a worked calculation is provided in Supplementary Table S7A.

#### 2.3.5 Probit transformation, regression diagnostic, and descriptive classification

For descriptive grading, the 16 WRSR values were sorted in ascending order. For the *i*-th distinct WRSR value, fi denotes its frequency and *Ci* denotes the cumulative count:

$$C_{i}=\overset{i}{\underset{\ell =1}{\sum }}\;f_{\ell }$$ (11)

The corresponding cumulative proportion was:

$$P_{i}=\frac{C_{i}}{m}$$ (12)

In the present data, all 16 WRSR values were distinct; therefore, *fi* = 1 and *Ci* was identical to the ascending rank of the WRSR value. For the maximum WRSR value, *Pi* would equal 1 and the corresponding standard normal quantile would be infinite. The terminal cumulative proportion was therefore corrected using the established RSR convention (15):

$$P_{m}^{\mathrm{corr}}=1-\frac{1}{4m}$$ (13)

Each cumulative proportion was transformed to a probability-unit score (Probit) as:

$$Y_{i}=5+\Phi^{-1}(P_{i})$$ (14)

where $\Phi^{-1}$ denotes the inverse standard normal cumulative distribution function. To evaluate the linear relation between the observed WRSR values and their normal probability scores, an ordinary least-squares regression model was fitted following standard RSR grading practice (16):

$$\mathrm{WRS}R_{i}=\beta_{0}+\beta_{1}Y_{i}+\varepsilon_{i}$$ (15)

where *ε_i_* is the residual term. The fitted equation and model diagnostics are reported in the Results and Supplementary Table S7B. This regression is a distributional diagnostic and does not alter the observed WRSR values or team rankings.

Four descriptive performance categories were defined on the Probit scale using *Y* = 4, 5, and 6 as cut points. These values correspond to cumulative probabilities of approximately 15.87%, 50.00%, and 84.13%, respectively. Probit values of 4 and 6 are commonly used as lower and upper grading nodes in RSR applications, including sports performance evaluation (15), while recent four-level RSR applications further divide the central band at *Y* = 5 (17). Accordingly, the categories were Poor (*Y* ≤ 4.0), Moderate (4.0 < *Y* ≤ 5.0), Good (5.0 < *Y* ≤ 6.0), and Excellent (*Y* > 6.0). These categories are sample-relative descriptive strata rather than externally validated performance standards. To assess the dependence of interpretation on this four-level choice, a three-level sensitivity classification was also examined: Poor (*Y* ≤ 4.0), Intermediate (4.0 < *Y* ≤ 6.0), and Excellent (*Y* > 6.0). The sensitivity analysis changes only category labels; WRSR values and ranks remain unchanged. The four-level criteria are summarized in Table 2. The complete mathematical formulation of the entropy-weighted WRSR and Probit procedure is presented in Equations 1–15.

**Table 2:** Four-level descriptive classification criteria on the probit scale.

Grade	Poor	Moderate	Good	Excellent
Probit value	*Y* ≤ 4.0	4.0 < *Y* ≤ 5.0	5.0 < *Y* ≤ 6.0	*Y* > 6.0
Approx. cumulative probability	≤15.87%	15.87%–50.00%	50.00%–84.13%	>84.13%

### 2.4 China-Australia comparison, final-match deviation, and cumulative-stage trajectory

After the comprehensive evaluation of the 16 teams, China and Australia were compared in terms of WRSR value, overall ranking, performance grade, and tournament-level differences in the 11 technical indicators. To further analyze performance changes in the final, pre-final means were used as the baseline to calculate final-match deviation. The pre-final mean did not include the final, thereby avoiding the influence of final-match performance on the baseline.

Deviation=[(finalvalue–pre-finalmean)/pre-finalmean]×100%

where the final value denotes a team's single-game technical performance in the China-Australia final and the pre-final mean denotes the team's average performance across all games before the final. For shooting percentages, the pre-final baseline was calculated from total made shots and attempts before the final. For the negative indicators fouls and turnovers, a positive deviation indicates an increase in unfavorable events and should therefore be interpreted in the opposite performance direction.

To examine how the comprehensive evaluation evolved across the tournament rather than relying only on the terminal ranking, an exploratory cumulative-stage WRSR trajectory analysis was additionally performed. Four prespecified snapshots were evaluated: the end of the group stage (through Game G24), the end of the quarter-final phase including qualification-to-quarter-finals and quarter-finals (through G32), the end of the semifinals (through G34), and tournament completion including the third-place game and final (through G36). At each snapshot, all team-game records available up to that point were re-aggregated for the same 16 teams using the indicator definitions described above. The full standardization, entropy-weight estimation, indicator ranking, and WRSR calculation pipeline was then re-estimated independently for that cumulative dataset. Teams already eliminated remained represented by their completed records, while teams still competing incorporated their newly available games. This cumulative design retained a constant set of 16 evaluation objects and avoided separate stage-only WRSR models in which the number and composition of teams would change sharply in later rounds. Interim Probit grades were not assigned because the purpose of this extension was to describe continuous changes in WRSR values and ranks across tournament progression. Full trajectories and stage-specific entropy weights are reported in Supplementary Tables S8, S9 and Supplementary Figure S2.

### 2.5 Statistical analysis

Data organization, internal consistency checks, team-level aggregation, indicator standardization, entropy-weight calculation, WRSR evaluation, final-match deviation analysis, and cumulative-stage WRSR recomputation were completed using Microsoft Excel. Pearson correlation analysis was performed using IBM SPSS Statistics 26.0 on tournament-level aggregated data from the 16 teams. Two-sided Pearson correlation coefficients (*r*) and corresponding *P* values were reported; *P* < 0.05 was considered statistically significant. Correlation analysis was used only to support assessment of information overlap among candidate indicators; final indicator retention also considered mathematical relationships, basketball-specific interpretability, and model requirements. Ordinary least-squares regression was used to quantify the relation between WRSR and Probit scores, with *R*^2^, *F*, and *P* reported as model diagnostics. A three-level Probit classification was examined as a sensitivity analysis of the four-level descriptive grading scheme. For the exploratory stage trajectory, the complete entropy-weighted WRSR pipeline was independently re-estimated at each cumulative tournament snapshot; no inferential test was applied to differences between snapshots. No hypothesis test was used to infer statistical significance between adjacent WRSR ranks, because WRSR is a relative composite ranking measure rather than an inferential test statistic. All calculations followed the indicator definitions and equations specified above. Results describe relative technical-performance structure and should not be interpreted as causal effects of individual indicators on match outcomes.

## 3 Results

### 3.1 Entropy weights and WRSR comprehensive evaluation

The entropy-weighted model produced indicator weights summing to 1.0000 (Table 3). Assists, steals, offensive rebounds, and free throw percentage received the largest weights (0.1216, 0.1191, 0.1141, and 0.1124, respectively), accounting for 46.72% cumulatively. Three-point field goal percentage had the smallest weight (0.0454). The full weight distribution is shown in Supplementary Figure S1.

**Table 3:** Entropy weights of the 11 technical indicators.

Rank	Indicator	Direction	Information entropy *e_j_*	Information utility *d_j_*	Entropy weight *w_j_*	Weight (%)
1	Assists	Positive	0.9068	0.0932	0.1216	12.16%
2	Steals	Positive	0.9087	0.0913	0.1191	11.91%
3	Offensive rebounds	Positive	0.9125	0.0875	0.1141	11.41%
4	Free throw percentage	Positive	0.9139	0.0861	0.1124	11.24%
5	Fouls	Negative	0.9288	0.0712	0.0928	9.28%
6	Blocks	Positive	0.9316	0.0684	0.0893	8.93%
7	Points	Positive	0.9320	0.0680	0.0887	8.87%
8	Defensive rebounds	Positive	0.9371	0.0629	0.0821	8.21%
9	2-point field goal percentage	Positive	0.9438	0.0562	0.0734	7.34%
10	Turnovers	Negative	0.9531	0.0469	0.0611	6.11%
11	3-point field goal percentage	Positive	0.9652	0.0348	0.0454	4.54%

The standardized matrix and proportion matrix are shown in Supplementary Table S5.

In the comprehensive evaluation, Australia ranked first (WRSR = 0.7714), followed by New Zealand (0.7262) and Japan (0.6844); all three were classified as Excellent under the four-level descriptive scheme (Table 4; Figure 1). China ranked seventh with a WRSR value of 0.5966 and was classified as Good.

**Table 4:** WRSR comprehensive evaluation ranking of the 16 teams.

Rank	Team	WRSR value	Probit value	Grade
1	Australia	0.7714	7.1539	Excellent
2	New Zealand	0.7262	6.5341	Excellent
3	Japan	0.6844	6.1503	Excellent
4	Korea	0.6747	5.8871	Good
5	Lebanon	0.6655	5.6745	Good
6	Philippines	0.6257	5.4888	Good
7	China	0.5966	5.3186	Good
8	Saudi Arabia	0.5347	5.1573	Good
9	India	0.4968	5.0000	Moderate
10	Iraq	0.4535	4.8427	Moderate
11	Jordan	0.4490	4.6814	Moderate
12	Iran	0.4251	4.5112	Moderate
13	Qatar	0.4190	4.3255	Moderate
14	Chinese Taipei	0.3940	4.1129	Moderate
15	Guam	0.3589	3.8497	Poor
16	Syria	0.2245	3.4659	Poor

Probit values were obtained from cumulative proportions after sorting WRSR values in ascending order. The maximum cumulative proportion was corrected as 1–1/(4 *m*), with *m* = 16. Grades are descriptive Probit-based strata and do not imply statistically significant differences between adjacent WRSR ranks. Indicator ranks and weighted rank contributions are shown in Supplementary Table S6; the worked calculation and sensitivity analysis are shown in Supplementary Table S7.

**Figure 1:**
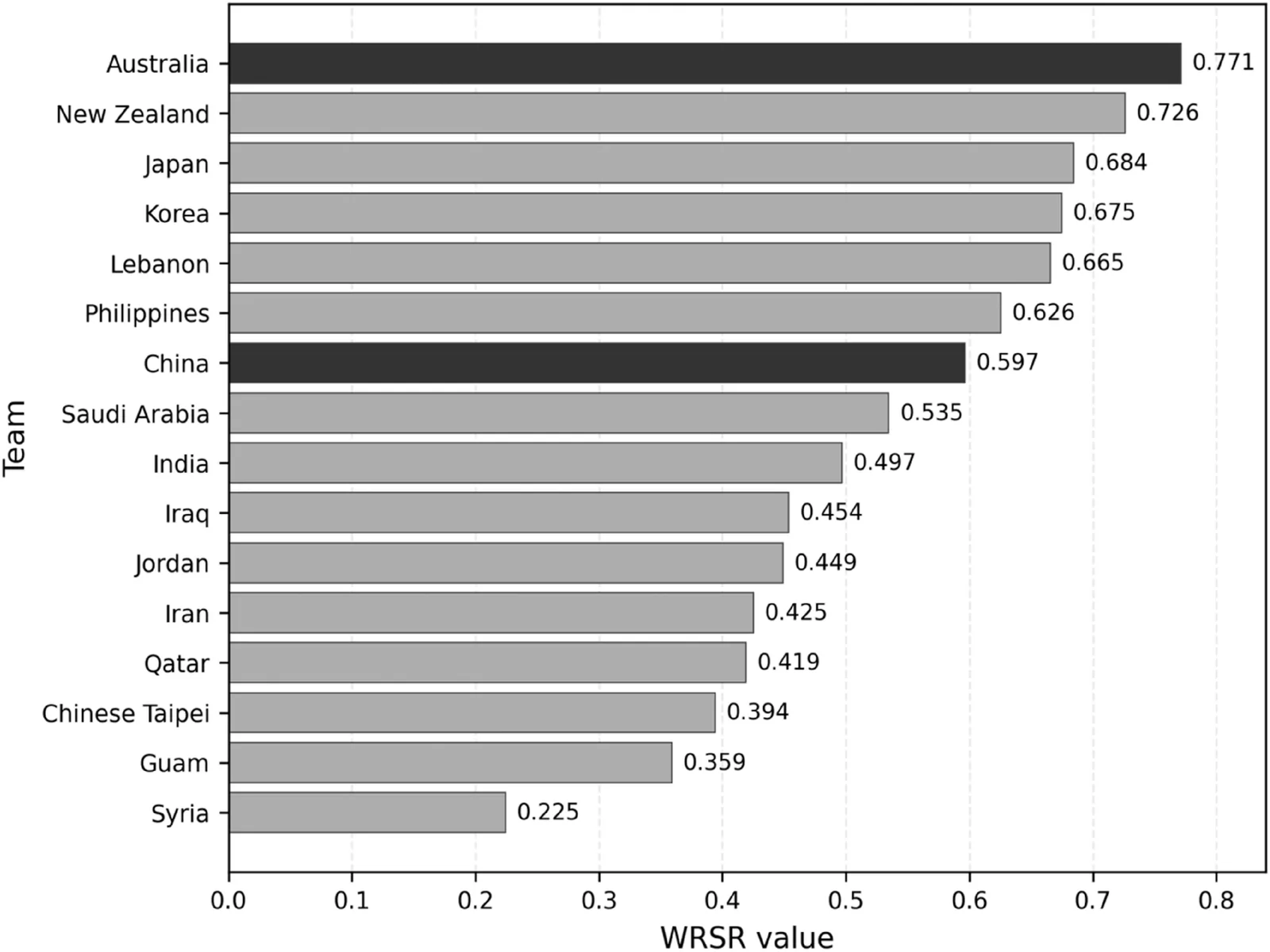
WRSR comprehensive evaluation ranking of the 16 teams. The *x*-axis shows WRSR values and the *y*-axis shows teams; the darker bars indicate China and Australia.

The WRSR values showed a strong linear relation with the Probit scores [fitted WRSR = −0.2378 + 0.1498 × Probit; *R*^2^ = 0.9524; *F*_(1,14)_ = 280.32; *P* < 0.001]. Under the alternative three-level sensitivity classification, Japan, New Zealand, and Australia remained in the upper category and Syria and Guam remained in the lower category, while the Moderate and Good groups were combined into a single intermediate category. WRSR values and team rankings were unchanged (Supplementary Table S7B).

### 3.2 Overall technical performance of China and Australia

As shown in Table 5 and Figure 2, Australia averaged 94.33 points per game, while China averaged 91.50 points. China's 2-point field goal percentage, 3-point field goal percentage, and free throw percentage were 55.5%, 39.9%, and 80.3%, respectively, while Australia's corresponding values were 55.1%, 38.9%, and 68.9%. Australia's offensive rebounds, defensive rebounds, steals, and blocks were 15.00, 33.00, 6.67, and 4.50 per game, respectively, while China's corresponding values were 12.50, 26.67, 4.33, and 1.17. China averaged 10.33 turnovers compared with Australia's 11.67, and China averaged 19.17 fouls compared with Australia's 18.83.

**Table 5:** Overall technical performance comparison between China and Australia.

Indicator	Direction	China	Australia	Difference (Australia-China)
Points	Positive	91.50	94.33	2.83
2-point field goal percentage	Positive	55.5%	55.1%	−0.3 percentage points
3-point field goal percentage	Positive	39.9%	38.9%	−1.0 percentage points
Free throw percentage	Positive	80.3%	68.9%	−11.4 percentage points
Offensive rebounds	Positive	12.50	15.00	2.50
Defensive rebounds	Positive	26.67	33.00	6.33
Assists	Positive	18.33	19.83	1.50
Fouls	Negative	19.17	18.83	−0.33
Turnovers	Negative	10.33	11.67	1.33
Steals	Positive	4.33	6.67	2.33
Blocks	Positive	1.17	4.50	3.33

For the negative indicators fouls and turnovers, lower values indicate a more favorable evaluation direction.

**Figure 2:**
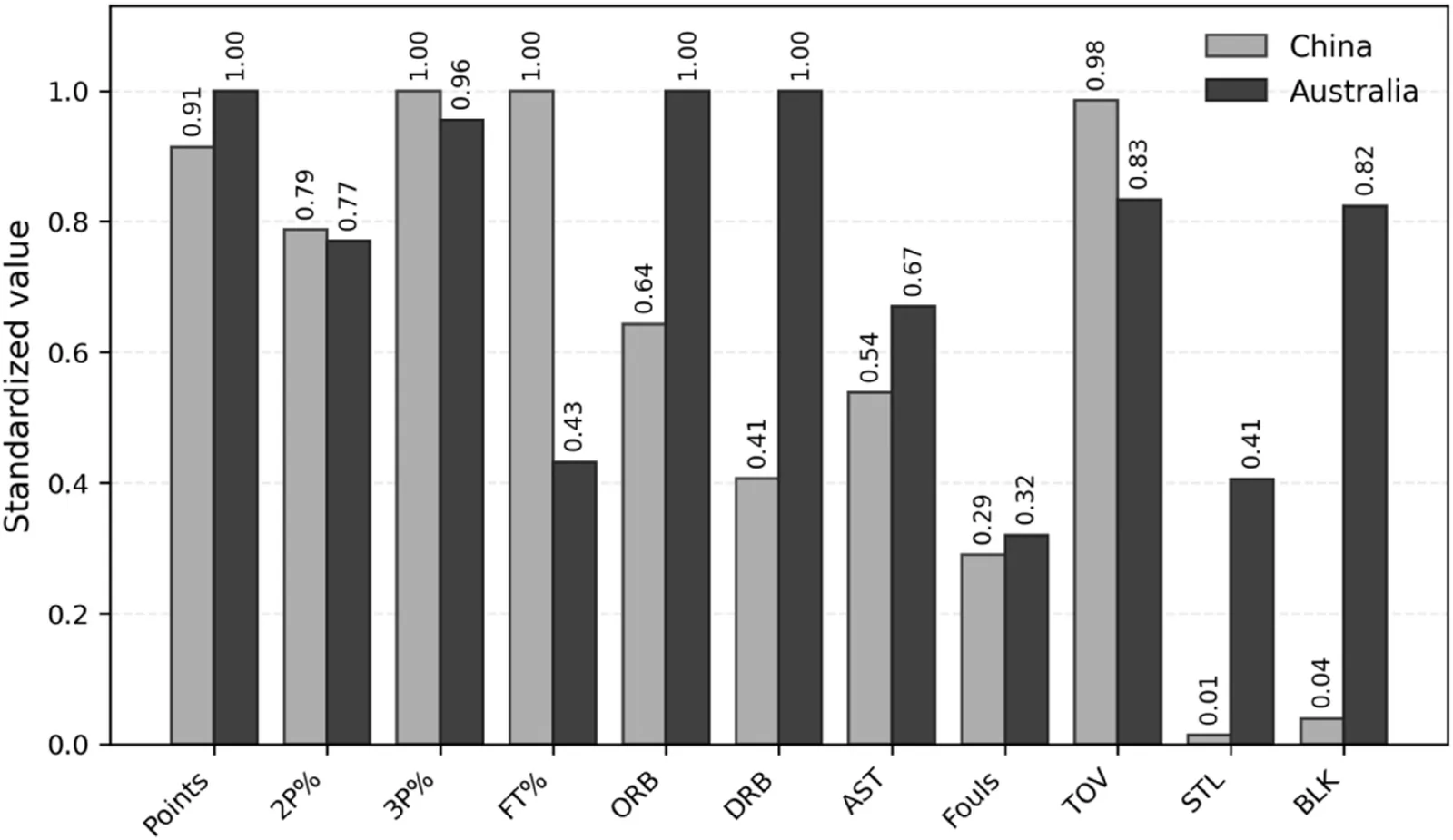
Comparison of standardized technical indicators between the Chinese and Australian teams. All indicators were standardized to 0–1 based on the 16-team sample; fouls and turnovers were reverse-coded as negative indicators.

### 3.3 Final-match deviation analysis of China and Australia

Final-match deviations were calculated using each team's pre-final mean as the baseline (Table 6 and Figure 3). China scored 89 points in the final, a 3.3% decrease from its pre-final mean, whereas Australia scored 90 points, a 5.5% decrease from its pre-final mean. China's 2-point field goal percentage and 3-point field goal percentage decreased by 5.3% and 19.1%, respectively, whereas its free throw percentage increased by 10.5%. Australia's 2-point field goal percentage increased by 5.2%, whereas its 3-point field goal percentage and free throw percentage decreased by 16.4% and 37.9%, respectively. China's offensive rebounds, assists, and steals decreased by 23.1%, 8.6%, and 34.8%, respectively. Australia's turnovers decreased by 53.1%, whereas assists and blocks increased by 19.8% and 42.9%, respectively.

**Table 6:** Final-match performance deviation analysis for China and Australia.

Indicator	China pre-final mean	China final value	China deviation	Australia pre-final mean	Australia final value	Australia deviation
Points	92.0	89.0	−3.3%	95.2	90.0	−5.5%
2-point field goal percentage	55.9%	52.9%	−5.3%	54.6%	57.4%	5.2%
3-point field goal percentage	41.2%	33.3%	−19.1%	39.8%	33.3%	−16.4%
Free throw percentage	78.4%	86.7%	10.5%	74.3%	46.2%	−37.9%
Offensive rebounds	13.0	10.0	−23.1%	15.0	15.0	0.0%
Defensive rebounds	26.6	27.0	1.5%	34.8	24.0	−31.0%
Assists	18.6	17.0	−8.6%	19.2	23.0	19.8%
Fouls	18.4	23.0	25.0%	18.8	19.0	1.1%
Turnovers	10.2	11.0	7.8%	12.8	6.0	−53.1%
Steals	4.6	3.0	−34.8%	7.0	5.0	−28.6%
Blocks	0.8	3.0	275.0%	4.2	6.0	42.9%

Deviations were calculated using pre-final means as the baseline; pre-final means did not include the final. Fouls and turnovers are negative indicators; positive deviations indicate increases in unfavorable events. The large deviation in China's blocks was related to its low pre-final baseline.

**Figure 3:**
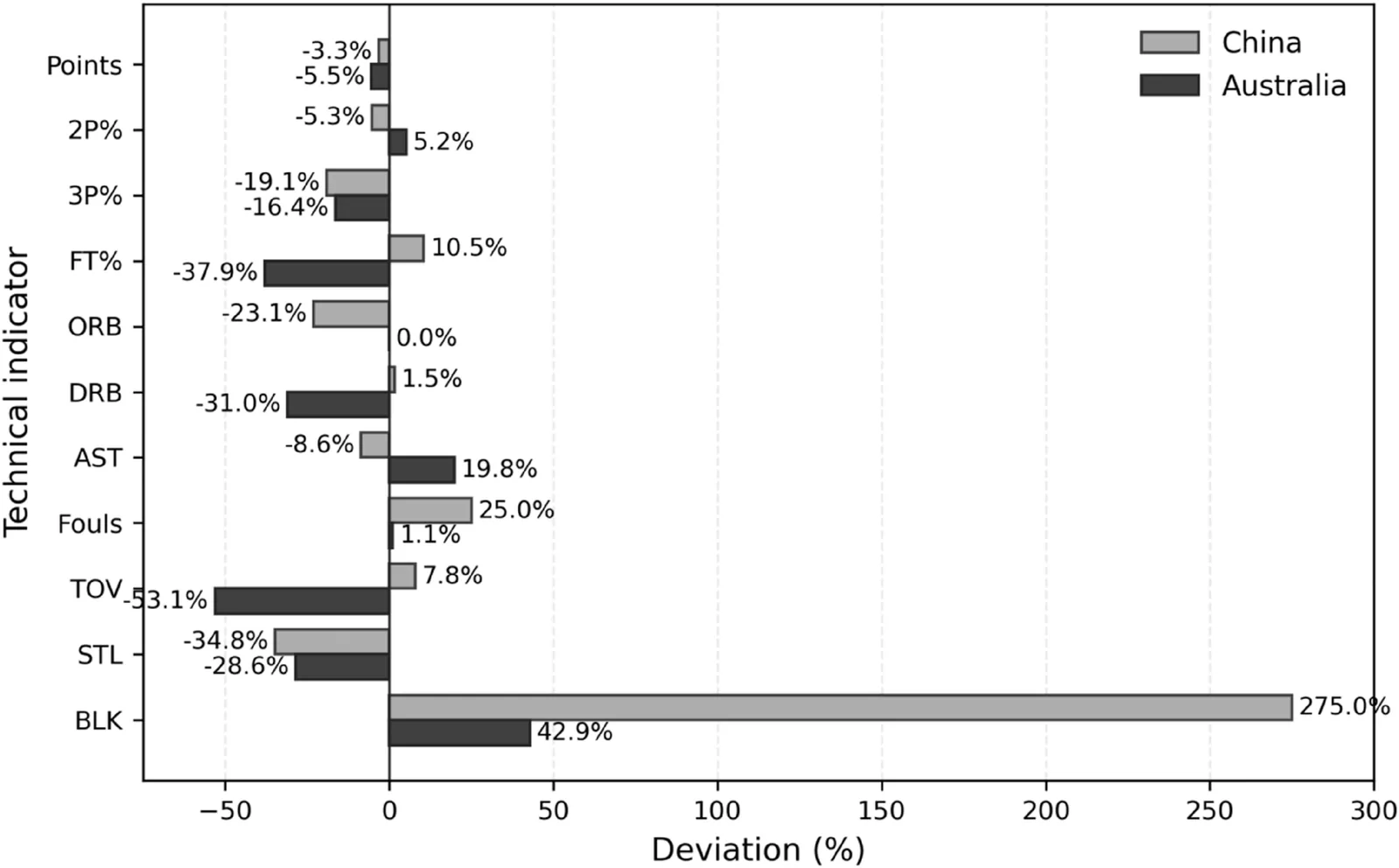
Comparison of final-match performance deviations between China and Australia. The *x*-axis shows deviation percentages, with 0% indicating the pre-final mean baseline; fouls and turnovers are negative indicators.

### 3.4 Cumulative-stage WRSR trajectories of China and Australia

The cumulative-stage analysis showed that Australia had a WRSR of 0.7884 (rank 2) at the end of the group stage, 0.7418 (rank 2) at the end of the quarter-final phase, 0.7585 (rank 1) after the semifinals, and 0.7714 (rank 1) at tournament completion. China moved from 0.6342 (rank 4) after the group stage to 0.6428 (rank 6) after the quarter-final phase, 0.6129 (rank 7) after the semifinals, and 0.5966 (rank 7) at tournament completion. Thus, Australia moved into first place by the semifinal snapshot and retained that position, whereas China shifted from fourth after the group stage to seventh by the semifinal snapshot and remained seventh in the final tournament evaluation. Because entropy weights and indicator ranks were re-estimated at each cumulative snapshot, the trajectory reflects the evolving information structure of the tournament rather than a fixed-weight projection. The complete 16-team trajectories, stage-specific entropy weights, and the China-Australia trajectory plot are provided in Supplementary Tables S8, S9 and Supplementary Figure S2.

This cumulative analysis is descriptive and should not be interpreted as an isolated effect of competition stage, because each snapshot retains all games played previously and teams differ in the number of games accumulated as the tournament progresses. Its purpose is to show how the same comprehensive evaluation framework changes as new tournament evidence becomes available.

## 4 Discussion

### 4.1 Main findings and interpretation of competitive performance

The tournament-level comparison showed that China and Australia reached the final with different multidimensional technical profiles. China recorded slightly higher 2-point, 3-point, and free throw percentages and fewer turnovers, whereas Australia recorded higher values for points, offensive and defensive rebounds, assists, steals, and blocks. These differences indicate that China's relative strengths were concentrated in shooting efficiency and possession stability, while Australia's profile was stronger in rebounding control, offensive organization, defensive pressure, and rim protection. This interpretation is consistent with basketball research showing that successful performance is typically associated with combinations of game-related statistics rather than a single indicator (18–24). Cabarkapa et al. (18), for example, identified shooting efficiency, defensive rebounding, assists, steals, blocks, and turnover control as discriminative statistics in NCAA Division I men's basketball. The present findings should nevertheless be interpreted as a descriptive tournament-wide profile rather than as evidence that any individual statistic caused match success. China's seventh-place WRSR ranking despite reaching the final further illustrates that knockout advancement and tournament-level composite evaluation capture related but distinct aspects of competitive performance. A team's progress in a knockout competition can also be influenced by opponent strength, schedule, game-day performance, and a small number of high-leverage possessions.

Entropy weighting further showed that assists, steals, offensive rebounds, and free throw percentage had the largest weights, accounting for 46.72% of the total. These weights quantify information discrimination among the 16 teams; they do not quantify causal importance for winning. Assists received the largest weight, indicating substantial between-team variation in ball movement and offensive organization within this tournament. Previous basketball research has repeatedly identified assists and related measures of coordinated offense as useful descriptors of team performance (25–28). The relatively high weights for steals and offensive rebounds likewise indicate that teams differed meaningfully in defensive disruption and second-chance opportunity creation. For teams with similar shooting efficiency, these dimensions can help differentiate how possessions are generated, extended, or disrupted, although the present model does not assign causal effects to them. Free throw percentage also showed relatively high discrimination across teams. Because it is an efficiency measure rather than a direct measure of contact frequency or offensive aggressiveness, its weight should be interpreted as reflecting between-team variation in free throw conversion within this sample. Overall, the weight distribution supports a multidimensional interpretation of tournament performance rather than a single-factor explanation.

The final-match deviation analysis provided a within-team complement to the tournament-level comparison. Relative to its pre-final baseline, China showed a higher free throw percentage but lower offensive rebounds, assists, and steals, together with increases in fouls and turnovers. Its 2-point and 3-point field goal percentages also declined, particularly from three-point range. Australia likewise showed mixed deviations: its free throw percentage and defensive rebounds decreased, whereas turnovers fell markedly and assists and blocks increased. These patterns suggest that the final differed from each team's preceding tournament profile in several dimensions, especially possession control, offensive organization, defensive pressure, and rim protection. Contextual factors such as schedule, travel demands, opponent quality, competition phase, and match outcome can influence basketball game-related statistics (29–32). Accordingly, the observed deviations should not be interpreted as changes in underlying team ability or as causal determinants of the one-point final. Instead, they identify the technical-statistical dimensions in which each finalist's performance departed most from its own pre-final reference. This baseline-based interpretation extends prior work using official FIBA statistics to describe performance differences across international competitions (33).

The cumulative-stage trajectory adds a temporal perspective to the end-of-tournament results. Australia was already near the top of the comprehensive ranking after the group stage and moved to first place by the semifinal snapshot, whereas China moved from fourth after the group stage to seventh as later-round games were incorporated. Previous basketball studies have shown that game-related statistics and their discriminative value can vary across regular-season, playoff, and finals contexts (29, 31, 32). The trajectory reported here should nevertheless be interpreted as an evolving cumulative evaluation rather than an estimate of an independent stage effect, because each snapshot retains all earlier games. Its practical value is to show whether a team's composite position remains relatively stable or changes as progressively higher-stakes tournament evidence is added.

### 4.2 Theoretical contributions and practical implications

This study makes two methodological contributions. First, it integrates indicator screening, entropy weighting, WRSR comprehensive evaluation, and final-match deviation analysis within a single tournament-level workflow. The framework therefore serves two related descriptive purposes: it locates each team within the competitive field and, for a selected key match, evaluates whether the observed technical profile departs from the team's own pre-match baseline. This combination adds a within-team reference to tournament-level comparison and reduces reliance on interpreting a final from one isolated box score. Second, the framework separates statistical discrimination from causal interpretation. Entropy weights quantify how strongly indicators differentiate teams in the observed sample, whereas WRSR values summarize relative comprehensive technical performance; neither quantity should be interpreted as the causal effect of an indicator on winning or as a direct prediction of the match result. This distinction is important for preserving the inferential boundaries of multidimensional descriptive models.

From a coaching and management perspective, post-match evaluation and real-time monitoring operate at different decision horizons and are therefore complementary rather than competing approaches. Real-time tracking, physiological monitoring, and technology-assisted feedback can support immediate observation and adjustment during training or competition (13, 14). The present framework addresses a different post-match question: whether a team's multidimensional technical profile in a key game was consistent with its established tournament baseline, and which dimensions showed the largest favorable or unfavorable deviations. This information can support structured debriefing, prioritization of subsequent training and scouting tasks, separation of persistent tournament-level tendencies from single-game fluctuations, and longitudinal benchmarking when consistent indicator definitions are retained. For China, the present results indicate that review should not focus only on shooting efficiency but should also consider offensive rebounding, ball movement, defensive pressure, and rim protection. Such findings can guide targeted review of relevant possessions and training tasks; however, box-score evaluation should inform rather than replace possession-level video analysis, tracking data, physiological monitoring, or coaching expertise. Its managerial value lies in converting standardized post-match statistics into an interpretable diagnostic layer that can be combined with faster and more context-rich information sources before the next match.

### 4.3 Limitations and future directions

This study has several limitations. First, the analysis relied on official FIBA Box Scores. These data are standardized and reproducible but do not capture process-level information such as game flow, shot location, tactical execution, spatial organization, or possession context. The findings therefore apply to team-level technical-statistical performance and should not be interpreted as direct evidence of tactical mechanisms. Second, no independent video coding was performed; interpretations of game context are based on the statistical profiles rather than possession-level video evidence. Third, the analysis did not distinguish individual players, lineup combinations, or within-game periods. Performance in key games may also be influenced by player availability, rotation structure, opponent matchups, and high-leverage possessions. Future studies could integrate possession-level video, player-tracking data, and contextual variables to address these mechanisms. Fourth, the study examined a single edition of the FIBA Men's Asia Cup, so the entropy weights and WRSR distributions are sample-specific and external generalizability is limited. Replication across FIBA Basketball World Cups, Olympic tournaments, continental championships, and multiple editions would allow assessment of temporal stability and cross-competition transportability. Finally, the cumulative-stage analysis uses progressively accumulated records rather than independent stage-only samples. It therefore describes how the tournament-level evaluation evolves as new games are added but cannot isolate the effect of a specific competition stage. Stage-specific inferential analyses would require larger multi-tournament samples and explicit adjustment for opponent strength, schedule, and other contextual factors.

## 5 Conclusion

This study developed a tournament-level team performance evaluation framework using official statistics from all 36 games of the 2025 FIBA Men's Asia Cup by integrating indicator screening, entropy weighting, WRSR comprehensive evaluation, and final-match deviation analysis. Assists, steals, offensive rebounds, and free throw percentage showed relatively high information discrimination among the 16 teams. Australia ranked first in the WRSR evaluation, whereas China ranked seventh. China showed relative strengths in shooting efficiency and turnover control, while Australia recorded higher values for points, rebounds, assists, steals, and blocks. The cumulative-stage analysis further showed Australia moving from rank 2 after the group stage to rank 1 by the semifinal snapshot, while China moved from rank 4 to rank 7 as later-round games were incorporated. Relative to pre-final baselines, China's offensive rebounds, assists, and steals decreased while fouls and turnovers increased; Australia showed more favorable deviations in turnovers, assists, and blocks. These findings describe multidimensional differences in technical-performance structure and key-match deviation rather than causal determinants of the final outcome. The framework is therefore best positioned as a reproducible post-match and between-match decision-support tool that complements, rather than replaces, real-time monitoring, possession-level analysis, and coaching judgment.

## Data Availability

The original contributions presented in the study are included in the article/Supplementary Material, further inquiries can be directed to the corresponding author.
