# Supplementary material for: Team performance evaluation in the 2025 FIBA Men’s Asia Cup: an entropy-weighted rank-sum ratio analysis with a case study of the China–Australia final
Source: Front Sports Act Living. 2026 Sep 15;8:1919832. doi: 10.3389/fspor.2026.1919832 (PMC13619583; doi:10.3389/fspor.2026.1919832)
Supplement: Supplementary file 1 [file Datasheet1.docx]

**Supplementary Material**

**Supplementary Table S1. Initial technical indicator pool and screening results**

| **Indicator category** | **Initial indicator** | **Decision** | **Rationale** |
| --- | --- | --- | --- |
| Scoring | Points | Retained | Reflects overall offensive output |
| Shooting | Field goals made | Excluded | Derived from or overlapping with field goal attempts, field goal percentage, and points |
| Shooting | Field goal attempts | Excluded | Derived from or overlapping with field goals made and field goal percentage |
| Shooting | Field goal percentage | Excluded | Can be further explained by 2-point and 3-point field goal percentages |
| Shooting | 2-point field goals made | Excluded | Overlaps with 2-point attempts and 2-point field goal percentage |
| Shooting | 2-point field goal attempts | Excluded | Reflects shot volume rather than efficiency and correlates with 2-point field goals made |
| Shooting | 2-point field goal percentage | Retained | Reflects mid-range and interior scoring efficiency |
| Shooting | 3-point field goals made | Excluded | Overlaps with 3-point attempts and 3-point field goal percentage |
| Shooting | 3-point field goal attempts | Excluded | Reflects shot volume rather than efficiency and correlates with 3-point field goals made |
| Shooting | 3-point field goal percentage | Retained | Reflects perimeter shooting efficiency |
| Free throws | Free throws made | Excluded | Derived from or overlapping with free throw attempts and free throw percentage |
| Free throws | Free throw attempts | Excluded | Reflects free throw opportunities and may double-count information from free throws made |
| Free throws | Free throw percentage | Retained | Reflects free throw efficiency |
| Rebounding | Total rebounds | Excluded | Composed of offensive and defensive rebounds |
| Rebounding | Offensive rebounds | Retained | Reflects ability to create second-chance opportunities |
| Rebounding | Defensive rebounds | Retained | Reflects defensive rebounding protection |
| Playmaking | Assists | Retained | Reflects team coordination and offensive organization |
| Discipline | Fouls | Retained | Reflects defensive discipline and foul control; negative indicator |
| Possession | Turnovers | Retained | Reflects possession stability; negative indicator |
| Defense | Steals | Retained | Reflects defensive pressure and transition-opportunity creation |
| Defense | Blocks | Retained | Reflects interior defensive impact |
| Composite efficiency | Efficiency value/rating | Excluded | Composite derived indicator that may overlap with multiple basic indicators |
| Outcome | Winning percentage/net point differential | Excluded | Outcome variable, not used as an explanatory variable for technical performance |

Note: The initial indicator pool contained 23 items. Retained indicators entered the subsequent entropy-weighted WRSR model; excluded indicators were not included in the final comprehensive evaluation.

**Supplementary Table S2. Correlation analysis and screening decisions for overlapping variables in the initial indicator pool**

| **Variable pair** | **Pearson r** | **P value** | **Use** |
| --- | --- | --- | --- |
| Field goals made-Field goal percentage | 0.899** | <0.001 | Used to support the assessment of information overlap or derivation |
| Field goal percentage-2-point field goal percentage | 0.918** | <0.001 | Used to support the assessment of information overlap or derivation |
| 2-point field goals made-2-point field goal attempts | 0.808** | <0.001 | Used to support the assessment of information overlap or derivation |
| 2-point field goals made-2-point field goal percentage | 0.546* | 0.029 | Used to support the assessment of information overlap or derivation |
| 3-point field goals made-3-point field goal attempts | 0.937** | <0.001 | Used to support the assessment of information overlap or derivation |
| 3-point field goals made-3-point field goal percentage | 0.901** | <0.001 | Used to support the assessment of information overlap or derivation |
| 3-point field goal attempts-3-point field goal percentage | 0.722** | 0.002 | Used to support the assessment of information overlap or derivation |
| Free throws made-Free throw attempts | 0.927** | <0.001 | Used to support the assessment of information overlap or derivation |
| Total rebounds-Offensive rebounds | 0.774** | <0.001 | Used to support the assessment of information overlap or derivation |
| Total rebounds-Defensive rebounds | 0.875** | <0.001 | Used to support the assessment of information overlap or derivation |
| Efficiency value-Points | 0.945** | <0.001 | Used to support the assessment of information overlap or derivation |
| Winning percentage-net point differential | 0.933** | <0.001 | Used to support the assessment of information overlap or derivation |

Note: The analysis was based on tournament-level aggregated data from the 16 participating teams. * P < 0.05, ** P < 0.01, two-sided tests.

**Supplementary Table S3. Correlation matrix among the 11 retained technical indicators**

| **Indicator** | **Points** | **2-point field goal percentage** | **3-point field goal percentage** | **Free throw percentage** | **Offensive rebounds** | **Defensive rebounds** | **Assists** | **Fouls** | **Turnovers** | **Steals** | **Blocks** |
| --- | --- | --- | --- | --- | --- | --- | --- | --- | --- | --- | --- |
| Points | 1 |  |  |  |  |  |  |  |  |  |  |
| 2-point field goal percentage | 0.822** | 1 |  |  |  |  |  |  |  |  |  |
| 3-point field goal percentage | 0.714** | 0.332 | 1 |  |  |  |  |  |  |  |  |
| Free throw percentage | 0.542* | 0.445 | 0.621* | 1 |  |  |  |  |  |  |  |
| Offensive rebounds | 0.532* | 0.364 | 0.287 | -0.170 | 1 |  |  |  |  |  |  |
| Defensive rebounds | 0.342 | 0.314 | 0.333 | -0.091 | 0.371 | 1 |  |  |  |  |  |
| Assists | 0.562* | 0.619* | 0.364 | 0.518* | 0.309 | 0.366 | 1 |  |  |  |  |
| Fouls | 0.507* | 0.418 | 0.277 | 0.206 | 0.041 | -0.107 | 0.079 | 1 |  |  |  |
| Turnovers | -0.545* | -0.339 | -0.489 | -0.413 | -0.323 | 0.194 | -0.443 | -0.264 | 1 |  |  |
| Steals | 0.249 | 0.258 | -0.105 | 0.058 | -0.005 | 0.042 | 0.365 | 0.388 | -0.030 | 1 |  |
| Blocks | 0.177 | 0.046 | 0.350 | 0.106 | 0.294 | 0.291 | 0.326 | 0.029 | -0.082 | -0.119 | 1 |

Note: * P < 0.05, ** P < 0.01.

**Supplementary Table S4. Raw data records, data completeness and internal consistency checks, and average technical indicators**

**Supplementary Table S4A. Seventy-two raw team-game records**

| **Game ID** | **Competition stage** | **Date** | **Team** | **Opponent** | **Points** | **2-point field goals made/attempted** | **3-point field goals made/attempted** | **Free throws made/attempted** | **Offensive rebounds** | **Defensive rebounds** | **Assists** | **Fouls** | **Turnovers** | **Steals** | **Blocks** | **FIBA Box Score URL** |
| --- | --- | --- | --- | --- | --- | --- | --- | --- | --- | --- | --- | --- | --- | --- | --- | --- |
| G01 | Group stage | 2025-08-05 | New Zealand | Iraq | 100 | 28/45 | 9/34 | 17/26 | 17 | 29 | 21 | 13 | 10 | 13 | 3 | https://www.fiba.basketball/en/events/fiba-asiacup-2025 |
| G01 | Group stage | 2025-08-05 | Iraq | New Zealand | 78 | 19/40 | 12/27 | 4/8 | 9 | 27 | 18 | 21 | 20 | 6 | 5 | https://www.fiba.basketball/en/events/fiba-asiacup-2025 |
| G02 | Group stage | 2025-08-05 | Jordan | India | 91 | 24/47 | 6/26 | 25/42 | 22 | 27 | 21 | 14 | 13 | 8 | 5 | https://www.fiba.basketball/en/events/fiba-asiacup-2025 |
| G02 | Group stage | 2025-08-05 | India | Jordan | 84 | 24/41 | 8/31 | 12/13 | 11 | 29 | 21 | 29 | 19 | 10 | 5 | https://www.fiba.basketball/en/events/fiba-asiacup-2025 |
| G03 | Group stage | 2025-08-05 | China | Saudi Arabia | 93 | 24/40 | 8/22 | 21/31 | 12 | 23 | 19 | 20 | 10 | 5 | 0 | https://www.fiba.basketball/en/events/fiba-asiacup-2025 |
| G03 | Group stage | 2025-08-05 | Saudi Arabia | China | 88 | 24/42 | 7/24 | 19/23 | 12 | 23 | 13 | 25 | 13 | 4 | 3 | https://www.fiba.basketball/en/events/fiba-asiacup-2025 |
| G04 | Group stage | 2025-08-06 | Chinese Taipei | Philippines | 95 | 16/34 | 12/27 | 27/35 | 6 | 18 | 12 | 28 | 4 | 6 | 2 | https://www.fiba.basketball/en/events/fiba-asiacup-2025 |
| G04 | Group stage | 2025-08-06 | Philippines | Chinese Taipei | 87 | 27/45 | 8/28 | 9/15 | 21 | 27 | 20 | 26 | 16 | 3 | 2 | https://www.fiba.basketball/en/events/fiba-asiacup-2025 |
| G05 | Group stage | 2025-08-06 | Australia | Korea | 97 | 21/44 | 15/26 | 10/14 | 14 | 35 | 13 | 13 | 17 | 7 | 4 | https://www.fiba.basketball/en/events/fiba-asiacup-2025 |
| G05 | Group stage | 2025-08-06 | Korea | Australia | 61 | 14/36 | 9/33 | 6/12 | 10 | 22 | 14 | 16 | 14 | 10 | 1 | https://www.fiba.basketball/en/events/fiba-asiacup-2025 |
| G06 | Group stage | 2025-08-06 | Japan | Syria | 99 | 23/40 | 14/32 | 11/16 | 11 | 30 | 22 | 18 | 10 | 9 | 4 | https://www.fiba.basketball/en/events/fiba-asiacup-2025 |
| G06 | Group stage | 2025-08-06 | Syria | Japan | 68 | 24/51 | 3/11 | 11/18 | 8 | 27 | 15 | 18 | 15 | 4 | 0 | https://www.fiba.basketball/en/events/fiba-asiacup-2025 |
| G07 | Group stage | 2025-08-06 | Guam | Iran | 52 | 18/42 | 3/22 | 7/11 | 9 | 28 | 4 | 14 | 10 | 3 | 3 | https://www.fiba.basketball/en/events/fiba-asiacup-2025 |
| G07 | Group stage | 2025-08-06 | Iran | Guam | 77 | 16/29 | 12/34 | 9/18 | 10 | 35 | 25 | 16 | 7 | 6 | 3 | https://www.fiba.basketball/en/events/fiba-asiacup-2025 |
| G08 | Group stage | 2025-08-07 | Qatar | Lebanon | 80 | 20/42 | 8/26 | 16/23 | 12 | 35 | 16 | 18 | 18 | 7 | 1 | https://www.fiba.basketball/en/events/fiba-asiacup-2025 |
| G08 | Group stage | 2025-08-07 | Lebanon | Qatar | 84 | 25/53 | 7/24 | 13/20 | 10 | 33 | 19 | 18 | 9 | 10 | 3 | https://www.fiba.basketball/en/events/fiba-asiacup-2025 |
| G09 | Group stage | 2025-08-07 | India | China | 69 | 22/43 | 6/20 | 7/11 | 8 | 17 | 16 | 11 | 12 | 7 | 6 | https://www.fiba.basketball/en/events/fiba-asiacup-2025 |
| G09 | Group stage | 2025-08-07 | China | India | 100 | 23/43 | 15/28 | 9/11 | 14 | 29 | 22 | 14 | 10 | 3 | 0 | https://www.fiba.basketball/en/events/fiba-asiacup-2025 |
| G10 | Group stage | 2025-08-07 | Iraq | Chinese Taipei | 60 | 20/44 | 3/21 | 11/15 | 13 | 28 | 16 | 17 | 21 | 6 | 1 | https://www.fiba.basketball/en/events/fiba-asiacup-2025 |
| G10 | Group stage | 2025-08-07 | Chinese Taipei | Iraq | 87 | 16/33 | 15/37 | 10/18 | 13 | 29 | 19 | 20 | 13 | 9 | 3 | https://www.fiba.basketball/en/events/fiba-asiacup-2025 |
| G11 | Group stage | 2025-08-07 | Philippines | New Zealand | 86 | 22/40 | 9/28 | 15/20 | 6 | 27 | 16 | 15 | 7 | 6 | 5 | https://www.fiba.basketball/en/events/fiba-asiacup-2025 |
| G11 | Group stage | 2025-08-07 | New Zealand | Philippines | 94 | 19/40 | 13/26 | 17/21 | 8 | 33 | 23 | 19 | 9 | 2 | 2 | https://www.fiba.basketball/en/events/fiba-asiacup-2025 |
| G12 | Group stage | 2025-08-08 | Saudi Arabia | Jordan | 77 | 14/30 | 11/32 | 16/24 | 11 | 27 | 22 | 19 | 15 | 6 | 8 | https://www.fiba.basketball/en/events/fiba-asiacup-2025 |
| G12 | Group stage | 2025-08-08 | Jordan | Saudi Arabia | 73 | 22/48 | 7/18 | 8/10 | 9 | 27 | 21 | 20 | 12 | 6 | 3 | https://www.fiba.basketball/en/events/fiba-asiacup-2025 |
| G13 | Group stage | 2025-08-08 | Korea | Qatar | 97 | 21/39 | 11/31 | 22/24 | 13 | 22 | 20 | 23 | 13 | 7 | 4 | https://www.fiba.basketball/en/events/fiba-asiacup-2025 |
| G13 | Group stage | 2025-08-08 | Qatar | Korea | 83 | 23/45 | 7/19 | 16/27 | 16 | 25 | 11 | 25 | 17 | 9 | 3 | https://www.fiba.basketball/en/events/fiba-asiacup-2025 |
| G14 | Group stage | 2025-08-08 | Iran | Japan | 78 | 24/46 | 7/20 | 9/16 | 6 | 26 | 16 | 23 | 8 | 12 | 2 | https://www.fiba.basketball/en/events/fiba-asiacup-2025 |
| G14 | Group stage | 2025-08-08 | Japan | Iran | 70 | 9/24 | 10/31 | 22/25 | 9 | 30 | 14 | 19 | 17 | 4 | 5 | https://www.fiba.basketball/en/events/fiba-asiacup-2025 |
| G15 | Group stage | 2025-08-08 | Lebanon | Australia | 80 | 24/49 | 4/22 | 20/27 | 12 | 30 | 18 | 23 | 11 | 8 | 3 | https://www.fiba.basketball/en/events/fiba-asiacup-2025 |
| G15 | Group stage | 2025-08-08 | Australia | Lebanon | 93 | 21/40 | 11/39 | 18/28 | 21 | 31 | 29 | 25 | 12 | 7 | 6 | https://www.fiba.basketball/en/events/fiba-asiacup-2025 |
| G16 | Group stage | 2025-08-09 | Syria | Guam | 73 | 29/47 | 3/19 | 6/8 | 9 | 25 | 16 | 14 | 16 | 8 | 3 | https://www.fiba.basketball/en/events/fiba-asiacup-2025 |
| G16 | Group stage | 2025-08-09 | Guam | Syria | 82 | 20/41 | 12/26 | 6/9 | 11 | 24 | 22 | 10 | 16 | 10 | 3 | https://www.fiba.basketball/en/events/fiba-asiacup-2025 |
| G17 | Group stage | 2025-08-09 | Iraq | Philippines | 57 | 11/34 | 9/26 | 8/11 | 7 | 38 | 14 | 11 | 16 | 5 | 7 | https://www.fiba.basketball/en/events/fiba-asiacup-2025 |
| G17 | Group stage | 2025-08-09 | Philippines | Iraq | 66 | 15/44 | 10/31 | 6/9 | 15 | 32 | 22 | 13 | 6 | 9 | 4 | https://www.fiba.basketball/en/events/fiba-asiacup-2025 |
| G18 | Group stage | 2025-08-09 | China | Jordan | 90 | 17/31 | 12/30 | 20/25 | 9 | 26 | 21 | 21 | 9 | 6 | 0 | https://www.fiba.basketball/en/events/fiba-asiacup-2025 |
| G18 | Group stage | 2025-08-09 | Jordan | China | 68 | 21/38 | 5/19 | 11/16 | 7 | 25 | 9 | 17 | 16 | 4 | 1 | https://www.fiba.basketball/en/events/fiba-asiacup-2025 |
| G19 | Group stage | 2025-08-09 | India | Saudi Arabia | 59 | 17/42 | 6/22 | 7/10 | 9 | 21 | 20 | 15 | 6 | 5 | 1 | https://www.fiba.basketball/en/events/fiba-asiacup-2025 |
| G19 | Group stage | 2025-08-09 | Saudi Arabia | India | 84 | 19/37 | 12/32 | 10/17 | 18 | 33 | 24 | 16 | 6 | 1 | 8 | https://www.fiba.basketball/en/events/fiba-asiacup-2025 |
| G20 | Group stage | 2025-08-10 | Chinese Taipei | New Zealand | 78 | 15/35 | 10/39 | 18/22 | 6 | 16 | 9 | 22 | 10 | 10 | 2 | https://www.fiba.basketball/en/events/fiba-asiacup-2025 |
| G20 | Group stage | 2025-08-10 | New Zealand | Chinese Taipei | 118 | 26/41 | 15/34 | 21/29 | 19 | 42 | 29 | 23 | 16 | 7 | 4 | https://www.fiba.basketball/en/events/fiba-asiacup-2025 |
| G21 | Group stage | 2025-08-10 | Qatar | Australia | 82 | 24/41 | 4/25 | 22/36 | 11 | 21 | 10 | 21 | 17 | 9 | 3 | https://www.fiba.basketball/en/events/fiba-asiacup-2025 |
| G21 | Group stage | 2025-08-10 | Australia | Qatar | 110 | 29/48 | 10/21 | 22/26 | 11 | 34 | 22 | 25 | 14 | 9 | 4 | https://www.fiba.basketball/en/events/fiba-asiacup-2025 |
| G22 | Group stage | 2025-08-10 | Guam | Japan | 63 | 14/44 | 9/25 | 8/16 | 11 | 22 | 13 | 10 | 10 | 7 | 0 | https://www.fiba.basketball/en/events/fiba-asiacup-2025 |
| G22 | Group stage | 2025-08-10 | Japan | Guam | 102 | 16/18 | 20/50 | 10/11 | 11 | 37 | 29 | 16 | 14 | 6 | 4 | https://www.fiba.basketball/en/events/fiba-asiacup-2025 |
| G23 | Group stage | 2025-08-10 | Korea | Lebanon | 97 | 12/26 | 22/38 | 7/13 | 8 | 23 | 29 | 14 | 13 | 9 | 2 | https://www.fiba.basketball/en/events/fiba-asiacup-2025 |
| G23 | Group stage | 2025-08-10 | Lebanon | Korea | 86 | 29/53 | 7/16 | 7/9 | 12 | 24 | 23 | 16 | 13 | 8 | 1 | https://www.fiba.basketball/en/events/fiba-asiacup-2025 |
| G24 | Group stage | 2025-08-11 | Syria | Iran | 43 | 15/42 | 1/11 | 10/18 | 7 | 25 | 9 | 17 | 19 | 4 | 0 | https://www.fiba.basketball/en/events/fiba-asiacup-2025 |
| G24 | Group stage | 2025-08-11 | Iran | Syria | 82 | 13/30 | 13/31 | 17/22 | 11 | 30 | 19 | 19 | 12 | 8 | 6 | https://www.fiba.basketball/en/events/fiba-asiacup-2025 |
| G25 | Qualification to Quarter-Finals | 2025-08-11 | Chinese Taipei | Jordan | 78 | 19/37 | 9/27 | 13/15 | 11 | 29 | 12 | 20 | 12 | 4 | 7 | https://www.fiba.basketball/en/events/fiba-asiacup-2025 |
| G25 | Qualification to Quarter-Finals | 2025-08-11 | Jordan | Chinese Taipei | 64 | 17/39 | 6/26 | 12/25 | 17 | 26 | 9 | 16 | 10 | 7 | 3 | https://www.fiba.basketball/en/events/fiba-asiacup-2025 |
| G26 | Qualification to Quarter-Finals | 2025-08-12 | Saudi Arabia | Philippines | 88 | 22/42 | 13/36 | 5/11 | 13 | 23 | 13 | 18 | 10 | 6 | 2 | https://www.fiba.basketball/en/events/fiba-asiacup-2025 |
| G26 | Qualification to Quarter-Finals | 2025-08-12 | Philippines | Saudi Arabia | 95 | 28/48 | 9/26 | 12/17 | 16 | 32 | 23 | 15 | 14 | 3 | 4 | https://www.fiba.basketball/en/events/fiba-asiacup-2025 |
| G27 | Qualification to Quarter-Finals | 2025-08-12 | Korea | Guam | 99 | 31/50 | 8/38 | 13/16 | 19 | 32 | 32 | 20 | 6 | 11 | 6 | https://www.fiba.basketball/en/events/fiba-asiacup-2025 |
| G27 | Qualification to Quarter-Finals | 2025-08-12 | Guam | Korea | 66 | 13/32 | 8/32 | 16/20 | 11 | 31 | 16 | 11 | 20 | 3 | 3 | https://www.fiba.basketball/en/events/fiba-asiacup-2025 |
| G28 | Qualification to Quarter-Finals | 2025-08-13 | Japan | Lebanon | 73 | 18/33 | 7/27 | 16/23 | 7 | 22 | 19 | 15 | 15 | 5 | 3 | https://www.fiba.basketball/en/events/fiba-asiacup-2025 |
| G28 | Qualification to Quarter-Finals | 2025-08-13 | Lebanon | Japan | 97 | 36/54 | 5/19 | 10/14 | 11 | 32 | 29 | 21 | 10 | 14 | 4 | https://www.fiba.basketball/en/events/fiba-asiacup-2025 |
| G29 | Quarter-Finals | 2025-08-13 | Australia | Philippines | 84 | 21/45 | 11/30 | 9/12 | 19 | 38 | 13 | 15 | 10 | 6 | 5 | https://www.fiba.basketball/en/events/fiba-asiacup-2025 |
| G29 | Quarter-Finals | 2025-08-13 | Philippines | Australia | 60 | 14/36 | 8/33 | 8/11 | 10 | 26 | 15 | 10 | 8 | 5 | 5 | https://www.fiba.basketball/en/events/fiba-asiacup-2025 |
| G30 | Quarter-Finals | 2025-08-14 | Iran | Chinese Taipei | 78 | 20/39 | 8/27 | 14/24 | 13 | 29 | 13 | 19 | 9 | 5 | 1 | https://www.fiba.basketball/en/events/fiba-asiacup-2025 |
| G30 | Quarter-Finals | 2025-08-14 | Chinese Taipei | Iran | 75 | 15/35 | 11/24 | 12/15 | 4 | 25 | 9 | 22 | 14 | 4 | 1 | https://www.fiba.basketball/en/events/fiba-asiacup-2025 |
| G31 | Quarter-Finals | 2025-08-14 | China | Korea | 79 | 24/49 | 7/25 | 10/13 | 20 | 29 | 16 | 21 | 9 | 2 | 2 | https://www.fiba.basketball/en/events/fiba-asiacup-2025 |
| G31 | Quarter-Finals | 2025-08-14 | Korea | China | 71 | 21/42 | 3/24 | 20/26 | 14 | 24 | 16 | 18 | 7 | 6 | 2 | https://www.fiba.basketball/en/events/fiba-asiacup-2025 |
| G32 | Quarter-Finals | 2025-08-15 | New Zealand | Lebanon | 90 | 25/39 | 7/27 | 19/25 | 4 | 31 | 19 | 13 | 14 | 12 | 4 | https://www.fiba.basketball/en/events/fiba-asiacup-2025 |
| G32 | Quarter-Finals | 2025-08-15 | Lebanon | New Zealand | 86 | 23/44 | 12/32 | 4/6 | 11 | 29 | 29 | 27 | 17 | 11 | 3 | https://www.fiba.basketball/en/events/fiba-asiacup-2025 |
| G33 | Semi-Finals | 2025-08-16 | China | New Zealand | 98 | 21/32 | 12/26 | 20/22 | 10 | 26 | 15 | 16 | 13 | 7 | 2 | https://www.fiba.basketball/en/events/fiba-asiacup-2025 |
| G33 | Semi-Finals | 2025-08-16 | New Zealand | China | 84 | 23/36 | 9/33 | 11/15 | 12 | 15 | 17 | 22 | 9 | 8 | 2 | https://www.fiba.basketball/en/events/fiba-asiacup-2025 |
| G34 | Semi-Finals | 2025-08-17 | Australia | Iran | 92 | 26/39 | 6/17 | 22/29 | 10 | 36 | 19 | 16 | 11 | 6 | 2 | https://www.fiba.basketball/en/events/fiba-asiacup-2025 |
| G34 | Semi-Finals | 2025-08-17 | Iran | Australia | 48 | 12/28 | 6/39 | 6/8 | 12 | 18 | 9 | 24 | 12 | 5 | 1 | https://www.fiba.basketball/en/events/fiba-asiacup-2025 |
| G35 | Third-place game | 2025-08-17 | Iran | New Zealand | 79 | 13/30 | 15/38 | 8/11 | 10 | 27 | 19 | 18 | 14 | 8 | 0 | https://www.fiba.basketball/en/events/fiba-asiacup-2025 |
| G35 | Third-place game | 2025-08-17 | New Zealand | Iran | 73 | 18/33 | 8/29 | 13/15 | 10 | 28 | 15 | 16 | 14 | 8 | 1 | https://www.fiba.basketball/en/events/fiba-asiacup-2025 |
| G36 | Final | 2025-08-18 | Australia | China | 90 | 27/47 | 8/24 | 12/26 | 15 | 24 | 23 | 19 | 6 | 5 | 6 | https://www.fiba.basketball/en/events/fiba-asiacup-2025 |
| G36 | Final | 2025-08-18 | China | Australia | 89 | 18/34 | 9/27 | 26/30 | 10 | 27 | 17 | 23 | 11 | 3 | 3 | https://www.fiba.basketball/en/events/fiba-asiacup-2025 |

Note: This table contains 72 raw team-game records. Game data were obtained from official FIBA Box Scores. Shooting percentages were calculated during aggregation by dividing tournament-level made shots by attempts.

**Supplementary Table S4B. Data completeness and internal consistency checks**

| **Check item** | **Check content** | **Result** |
| --- | --- | --- |
| Game completeness | All 36 games were included | Passed |
| Record completeness | Each game corresponded to two team records, giving 72 records in total | Passed |
| Game ID consistency | Each Game ID corresponded to two teams | Passed |
| Team-opponent mirror check | The two teams and opponents corresponded within the same game | Passed |
| Score mirror check | One team's score equaled the opponent score in the paired record | Passed |
| Score decomposition check | 2-point FGM x 2 + 3-point FGM x 3 + FTM equaled team points | Passed |
| Shooting percentage consistency check | Shooting percentages equaled the corresponding made shots divided by attempts | Passed |
| Model-eligible records | Records passing all checks | 72 records |

**Supplementary Table S4C. Average technical indicators of the 16 teams**

| **Team** | **Games** | **Points per game** | **2-point field goal percentage** | **3-point field goal percentage** | **Free throw percentage** | **Offensive rebounds per game** | **Defensive rebounds per game** | **Assists per game** | **Fouls per game** | **Turnovers per game** | **Steals per game** | **Blocks per game** |
| --- | --- | --- | --- | --- | --- | --- | --- | --- | --- | --- | --- | --- |
| Australia | 6 | 94.33 | 55.1% | 38.9% | 68.9% | 15 | 33 | 19.83 | 18.83 | 11.67 | 6.67 | 4.50 |
| China | 6 | 91.50 | 55.5% | 39.9% | 80.3% | 12.50 | 26.67 | 18.33 | 19.17 | 10.33 | 4.33 | 1.17 |
| New Zealand | 6 | 93.17 | 59.4% | 33.3% | 74.8% | 11.67 | 29.67 | 20.67 | 17.67 | 12 | 8.33 | 2.67 |
| Iraq | 3 | 65 | 42.4% | 32.4% | 67.6% | 9.67 | 31 | 16 | 16.33 | 19 | 5.67 | 4.33 |
| Jordan | 4 | 74 | 48.8% | 27.0% | 60.2% | 13.75 | 26.25 | 15 | 16.75 | 12.75 | 6.25 | 3 |
| India | 3 | 70.67 | 50.0% | 27.4% | 76.5% | 9.33 | 22.33 | 19 | 18.33 | 12.33 | 7.33 | 4 |
| Saudi Arabia | 4 | 84.25 | 52.3% | 34.7% | 66.7% | 13.50 | 26.50 | 18 | 19.50 | 11 | 4.25 | 5.25 |
| Chinese Taipei | 5 | 82.60 | 46.6% | 37.0% | 76.2% | 8 | 23.40 | 12.20 | 22.40 | 10.60 | 6.60 | 3 |
| Philippines | 5 | 78.80 | 49.8% | 30.1% | 69.4% | 13.60 | 28.80 | 19.20 | 15.80 | 10.20 | 5.20 | 4 |
| Korea | 5 | 85 | 51.3% | 32.3% | 74.7% | 12.80 | 24.60 | 22.20 | 18.20 | 10.60 | 8.60 | 3 |
| Japan | 4 | 86 | 57.4% | 36.4% | 78.7% | 9.50 | 29.75 | 21 | 17 | 14 | 6 | 4 |
| Syria | 3 | 61.33 | 48.6% | 17.1% | 61.4% | 8 | 25.67 | 13.33 | 16.33 | 16.67 | 5.33 | 1 |
| Guam | 4 | 65.75 | 40.9% | 30.5% | 66.1% | 10.50 | 26.25 | 13.75 | 11.25 | 14 | 5.75 | 2.25 |
| Iran | 6 | 73.67 | 48.5% | 32.3% | 63.6% | 10.33 | 27.50 | 16.83 | 19.83 | 10.33 | 7.33 | 2.17 |
| Qatar | 3 | 81.67 | 52.3% | 27.1% | 62.8% | 13 | 27 | 12.33 | 21.33 | 17.33 | 8.33 | 2.33 |
| Lebanon | 5 | 86.60 | 54.2% | 31.0% | 71.1% | 11.20 | 29.60 | 23.60 | 21 | 12 | 10.20 | 2.80 |

Note: Shooting percentages were calculated as total made shots divided by total attempts at the tournament level. All count-type indicators other than shooting percentages are reported as per-game values.

**Supplementary Figure S1. Entropy weight distribution of the 11 technical indicators**

Note: The x-axis shows entropy weight values and the y-axis shows technical indicators. Fouls and turnovers are negative indicators.

**Supplementary Table S5. Standardized matrix and proportion matrix for the 16 teams**

Note: Range standardization was used and indicator directions were unified. Fouls and turnovers were treated as negative indicators and reverse-coded.

**Supplementary Table S5A. Standardized indicator matrix for the 16 teams**

| **Team** | **Points** | **2-point field goal percentage** | **3-point field goal percentage** | **Free throw percentage** | **Offensive rebounds** | **Defensive rebounds** | **Assists** | **Fouls** | **Turnovers** | **Steals** | **Blocks** |
| --- | --- | --- | --- | --- | --- | --- | --- | --- | --- | --- | --- |
| Australia | 1.0000 | 0.7695 | 0.9553 | 0.4318 | 1.0000 | 1.0000 | 0.6696 | 0.3199 | 0.8333 | 0.4062 | 0.8235 |
| China | 0.9141 | 0.7871 | 1.0000 | 1.0000 | 0.6429 | 0.4062 | 0.5380 | 0.2900 | 0.9848 | 0.0140 | 0.0392 |
| New Zealand | 0.9646 | 1.0000 | 0.7132 | 0.7265 | 0.5238 | 0.6875 | 0.7427 | 0.4245 | 0.7955 | 0.6863 | 0.3922 |
| Iraq | 0.1111 | 0.0806 | 0.6736 | 0.3700 | 0.2381 | 0.8125 | 0.3333 | 0.5441 | 0.0000 | 0.2381 | 0.7843 |
| Jordan | 0.3838 | 0.4296 | 0.4339 | 0.0000 | 0.8214 | 0.3672 | 0.2456 | 0.5067 | 0.7102 | 0.3361 | 0.4706 |
| India | 0.2828 | 0.4924 | 0.4528 | 0.8092 | 0.1905 | 0.0000 | 0.5965 | 0.3647 | 0.7576 | 0.5182 | 0.7059 |
| Saudi Arabia | 0.6944 | 0.6175 | 0.7721 | 0.3212 | 0.7857 | 0.3906 | 0.5088 | 0.2601 | 0.9091 | 0.0000 | 1.0000 |
| Chinese Taipei | 0.6444 | 0.3062 | 0.8745 | 0.7953 | 0.0000 | 0.1000 | 0.0000 | 0.0000 | 0.9545 | 0.3950 | 0.4706 |
| Philippines | 0.5293 | 0.4797 | 0.5730 | 0.4594 | 0.8000 | 0.6062 | 0.6140 | 0.5919 | 1.0000 | 0.1597 | 0.7059 |
| Korea | 0.7172 | 0.5623 | 0.6686 | 0.7223 | 0.6857 | 0.2125 | 0.8772 | 0.3767 | 0.9545 | 0.7311 | 0.4706 |
| Japan | 0.7475 | 0.8915 | 0.8489 | 0.9185 | 0.2143 | 0.6953 | 0.7719 | 0.4843 | 0.5682 | 0.2941 | 0.7059 |
| Syria | 0.0000 | 0.4152 | 0.0000 | 0.0572 | 0.0000 | 0.3125 | 0.0994 | 0.5441 | 0.2652 | 0.1821 | 0.0000 |
| Guam | 0.1338 | 0.0000 | 0.5878 | 0.2915 | 0.3571 | 0.3672 | 0.1360 | 1.0000 | 0.5682 | 0.2521 | 0.2941 |
| Iran | 0.3737 | 0.4122 | 0.6667 | 0.1703 | 0.3333 | 0.4844 | 0.4064 | 0.2302 | 0.9848 | 0.5182 | 0.2745 |
| Qatar | 0.6162 | 0.6189 | 0.4416 | 0.1282 | 0.7143 | 0.4375 | 0.0117 | 0.0957 | 0.1894 | 0.6863 | 0.3137 |
| Lebanon | 0.7657 | 0.7165 | 0.6097 | 0.5395 | 0.4571 | 0.6813 | 1.0000 | 0.1256 | 0.7955 | 1.0000 | 0.4235 |

**Supplementary Table S5B. Indicator proportion matrix for the 16 teams**

| **Team** | **Points_pij** | **2-point field goal percentage_pij** | **3-point field goal percentage_pij** | **Free throw percentage_pij** | **Offensive rebounds_pij** | **Defensive rebounds_pij** | **Assists_pij** | **Fouls_pij** | **Turnovers_pij** | **Steals_pij** | **Blocks_pij** |
| --- | --- | --- | --- | --- | --- | --- | --- | --- | --- | --- | --- |
| Australia | 0.112628 | 0.089697 | 0.092999 | 0.055780 | 0.128795 | 0.132259 | 0.088674 | 0.051942 | 0.073937 | 0.063291 | 0.104582 |
| China | 0.102958 | 0.091745 | 0.097354 | 0.129182 | 0.082797 | 0.053730 | 0.071249 | 0.047087 | 0.087380 | 0.002183 | 0.004980 |
| New Zealand | 0.108646 | 0.116561 | 0.069429 | 0.093852 | 0.067464 | 0.090928 | 0.098354 | 0.068932 | 0.070576 | 0.106940 | 0.049801 |
| Iraq | 0.012514 | 0.009392 | 0.065582 | 0.047794 | 0.030666 | 0.107460 | 0.044143 | 0.088349 | 0.000000 | 0.037102 | 0.099602 |
| Jordan | 0.043231 | 0.050074 | 0.042242 | 0.000000 | 0.105796 | 0.048564 | 0.032527 | 0.082282 | 0.063015 | 0.052379 | 0.059761 |
| India | 0.031854 | 0.057392 | 0.044083 | 0.104536 | 0.024532 | 0.000000 | 0.078993 | 0.059223 | 0.067216 | 0.080751 | 0.089641 |
| Saudi Arabia | 0.078214 | 0.071980 | 0.075168 | 0.041489 | 0.101196 | 0.051664 | 0.067377 | 0.042233 | 0.080659 | 0.000000 | 0.126992 |
| Chinese Taipei | 0.072582 | 0.035691 | 0.085140 | 0.102735 | 0.000000 | 0.013226 | 0.000000 | 0.000000 | 0.084692 | 0.061545 | 0.059761 |
| Philippines | 0.059613 | 0.055915 | 0.055781 | 0.059353 | 0.103036 | 0.080182 | 0.081317 | 0.096116 | 0.088725 | 0.024880 | 0.089641 |
| Korea | 0.080774 | 0.065544 | 0.065089 | 0.093313 | 0.088316 | 0.028105 | 0.116166 | 0.061165 | 0.084692 | 0.113924 | 0.059761 |
| Japan | 0.084187 | 0.103908 | 0.082645 | 0.118659 | 0.027599 | 0.091961 | 0.102226 | 0.078641 | 0.050412 | 0.045832 | 0.089641 |
| Syria | 0.000000 | 0.048402 | 0.000000 | 0.007386 | 0.000000 | 0.041331 | 0.013166 | 0.088349 | 0.023526 | 0.028372 | 0.000000 |
| Guam | 0.015074 | 0.000000 | 0.057229 | 0.037661 | 0.045998 | 0.048564 | 0.018006 | 0.162378 | 0.050412 | 0.039284 | 0.037351 |
| Iran | 0.042093 | 0.048046 | 0.064910 | 0.022002 | 0.042932 | 0.064063 | 0.053824 | 0.037379 | 0.087380 | 0.080751 | 0.034861 |
| Qatar | 0.069397 | 0.072142 | 0.042996 | 0.016564 | 0.091996 | 0.057863 | 0.001549 | 0.015534 | 0.016804 | 0.106940 | 0.039841 |
| Lebanon | 0.086234 | 0.083511 | 0.059352 | 0.069694 | 0.058878 | 0.090101 | 0.132430 | 0.020388 | 0.070576 | 0.155827 | 0.053785 |

**Supplementary Table S6. Indicator rank matrix and weighted rank-contribution matrix for the 16 teams**

Note: For positive indicators, larger values received higher ranks; for the negative indicators fouls and turnovers, smaller values received higher ranks. Tied values were assigned average ranks.

**Supplementary Table S6A. Indicator rank matrix for the 16 teams**

| **Team** | **Points** | **2-point field goal percentage** | **3-point field goal percentage** | **Free throw percentage** | **Offensive rebounds** | **Defensive rebounds** | **Assists** | **Fouls** | **Turnovers** | **Steals** | **Blocks** |
| --- | --- | --- | --- | --- | --- | --- | --- | --- | --- | --- | --- |
| Australia | 16 | 13 | 15 | 8 | 16 | 16 | 12 | 7 | 10 | 10 | 15 |
| New Zealand | 15 | 16 | 11 | 12 | 9 | 13 | 13 | 10 | 8.5 | 13.5 | 6 |
| Japan | 12 | 15 | 13 | 15 | 4 | 14 | 14 | 11 | 4.5 | 7 | 12 |
| Korea | 11 | 9 | 9 | 11 | 11 | 3 | 15 | 9 | 12.5 | 15 | 9 |
| Lebanon | 13 | 12 | 7 | 10 | 8 | 12 | 16 | 3 | 8.5 | 16 | 7 |
| Philippines | 7 | 7 | 5 | 9 | 14 | 11 | 11 | 15 | 16 | 3 | 12 |
| China | 14 | 14 | 16 | 16 | 10 | 8 | 9 | 6 | 14.5 | 2 | 2 |
| Saudi Arabia | 10 | 10 | 12 | 6 | 13 | 7 | 8 | 5 | 11 | 1 | 16 |
| India | 4 | 8 | 4 | 14 | 3 | 1 | 10 | 8 | 7 | 11.5 | 12 |
| Iraq | 2 | 2 | 10 | 7 | 5 | 15 | 6 | 13.5 | 1 | 5 | 14 |
| Jordan | 6 | 6 | 2 | 1 | 15 | 5.5 | 5 | 12 | 6 | 8 | 9 |
| Iran | 5 | 4 | 8 | 4 | 6 | 10 | 7 | 4 | 14.5 | 11.5 | 3 |
| Qatar | 8 | 11 | 3 | 3 | 12 | 9 | 2 | 2 | 2 | 13.5 | 5 |
| Chinese Taipei | 9 | 3 | 14 | 13 | 1.5 | 2 | 1 | 1 | 12.5 | 9 | 9 |
| Guam | 3 | 1 | 6 | 5 | 7 | 5.5 | 4 | 16 | 4.5 | 6 | 4 |
| Syria | 1 | 5 | 1 | 2 | 1.5 | 4 | 3 | 13.5 | 3 | 4 | 1 |

**Supplementary Table S6B. Weighted rank-contribution matrix for the 16 teams**

| **Team** | **Points** | **2-point field goal percentage** | **3-point field goal percentage** | **Free throw percentage** | **Offensive rebounds** | **Defensive rebounds** | **Assists** | **Fouls** | **Turnovers** | **Steals** | **Blocks** | **Weighted rank sum** | **WRSR value** |
| --- | --- | --- | --- | --- | --- | --- | --- | --- | --- | --- | --- | --- | --- |
| Australia | 1.4197 | 0.9536 | 0.6809 | 0.8989 | 1.8260 | 1.3135 | 1.4589 | 0.6499 | 0.6114 | 1.1911 | 1.3391 | 12.3430 | 0.7714 |
| New Zealand | 1.3310 | 1.1737 | 0.4993 | 1.3483 | 1.0271 | 1.0672 | 1.5804 | 0.9284 | 0.5197 | 1.6080 | 0.5356 | 11.6189 | 0.7262 |
| Japan | 1.0648 | 1.1003 | 0.5901 | 1.6854 | 0.4565 | 1.1493 | 1.7020 | 1.0213 | 0.2751 | 0.8338 | 1.0713 | 10.9499 | 0.6844 |
| Korea | 0.9761 | 0.6602 | 0.4085 | 1.2359 | 1.2554 | 0.2463 | 1.8236 | 0.8356 | 0.7643 | 1.7867 | 0.8035 | 10.7960 | 0.6747 |
| Lebanon | 1.1535 | 0.8802 | 0.3177 | 1.1236 | 0.9130 | 0.9851 | 1.9452 | 0.2785 | 0.5197 | 1.9058 | 0.6249 | 10.6474 | 0.6655 |
| Philippines | 0.6211 | 0.5135 | 0.2270 | 1.0112 | 1.5978 | 0.9031 | 1.3373 | 1.3926 | 0.9783 | 0.3573 | 1.0713 | 10.0104 | 0.6257 |
| China | 1.2423 | 1.0269 | 0.7263 | 1.7977 | 1.1413 | 0.6568 | 1.0942 | 0.5571 | 0.8866 | 0.2382 | 0.1785 | 9.5457 | 0.5966 |
| Saudi Arabia | 0.8873 | 0.7335 | 0.5447 | 0.6741 | 1.4836 | 0.5747 | 0.9726 | 0.4642 | 0.6726 | 0.1191 | 1.4284 | 8.5548 | 0.5347 |
| India | 0.3549 | 0.5868 | 0.1816 | 1.5730 | 0.3424 | 0.0821 | 1.2157 | 0.7427 | 0.4280 | 1.3698 | 1.0713 | 7.9484 | 0.4968 |
| Iraq | 0.1775 | 0.1467 | 0.4539 | 0.7865 | 0.5706 | 1.2314 | 0.7294 | 1.2534 | 0.0611 | 0.5956 | 1.2498 | 7.2560 | 0.4535 |
| Jordan | 0.5324 | 0.4401 | 0.0908 | 0.1124 | 1.7119 | 0.4515 | 0.6079 | 1.1141 | 0.3669 | 0.9529 | 0.8035 | 7.1843 | 0.4490 |
| Iran | 0.4437 | 0.2934 | 0.3631 | 0.4494 | 0.6848 | 0.8210 | 0.8510 | 0.3714 | 0.8866 | 1.3698 | 0.2678 | 6.8019 | 0.4251 |
| Qatar | 0.7099 | 0.8069 | 0.1362 | 0.3371 | 1.3695 | 0.7389 | 0.2431 | 0.1857 | 0.1223 | 1.6080 | 0.4464 | 6.7039 | 0.4190 |
| Chinese Taipei | 0.7986 | 0.2201 | 0.6355 | 1.4606 | 0.1712 | 0.1642 | 0.1216 | 0.0928 | 0.7643 | 1.0720 | 0.8035 | 6.3043 | 0.3940 |
| Guam | 0.2662 | 0.0734 | 0.2723 | 0.5618 | 0.7989 | 0.4515 | 0.4863 | 1.4855 | 0.2751 | 0.7147 | 0.3571 | 5.7428 | 0.3589 |
| Syria | 0.0887 | 0.3668 | 0.0454 | 0.2247 | 0.1712 | 0.3284 | 0.3647 | 1.2534 | 0.1834 | 0.4765 | 0.0893 | 3.5924 | 0.2245 |

**Supplementary Table S7. Worked computational example and WRSR–Probit sensitivity analysis**

**Supplementary Table S7A. Step-by-step worked example using Australia**

| **Equation/step** | **Example object** | **Substitution** | **Computed value** |
| --- | --- | --- | --- |
| (1) Raw matrix | Australia—Points | x = 94.3333 points/game | 94.3333 |
| (2) Positive standardization | Australia—Points | (94.3333 - 61.3333) / (94.3333 - 61.3333) | 1.0000 |
| (3) Negative standardization | Australia—Turnovers | (19.0000 - 11.6667) / (19.0000 - 10.2000) | 0.8333 |
| (4) Standardized matrix | Australia—Points | Entry in Z equals standardized z value | 1.0000 |
| (5) Indicator proportion | Australia—Points | 1.0000 / Σz = 1.0000 / 8.8788 | 0.112628 |
| (6) Entropy constant | All indicators | 1 / ln(16) | 0.360674 |
| (7) Information entropy | Points | -0.360674 × Σ(p ln p), with Σ(p ln p) = -2.584007 | 0.931984 |
| (8) Information utility | Points | 1 - 0.931984 | 0.068016 |
| (9) Entropy weight | Points | 0.068016 / Σd (0.766536) | 0.088732 |
| (10) WRSR | Australia | Σ(wjRij) / 16 = 12.343009 / 16 | 0.771438 |
| (11) Cumulative count | Australia | Highest WRSR; all WRSR values are distinct, so f = 1 and C = 16 | 16 |
| (12) Cumulative proportion | Australia | C / m = 16 / 16 = 1.0000 before terminal correction | 1.000000 |
| (13) Terminal correction | Australia | 1 - 1 / (4 × 16) | 0.984375 |
| (14) Probit | Australia | 5 + Φ⁻¹(0.984375) | 7.153875 |
| (15) Regression diagnostic | Australia | 0.771438 = -0.237850 + 0.149787 × 7.153875 + ε | Fitted = 0.833709; ε = -0.062271 |

Note: Values were recomputed directly from the 72 team-game records. The worked example demonstrates every equation used in Section 2.3. Small differences in displayed decimals may arise from rounding; model calculations used unrounded values.

**Supplementary Table S7B. WRSR distribution, Probit transformation, regression diagnostics, and classification sensitivity**

| **Team** | **WRSR** | **Ascending rank q** | **P** | **Probit Y** | **Fitted WRSR** | **Residual** | **Four-level grade** | **Three-level grade** |
| --- | --- | --- | --- | --- | --- | --- | --- | --- |
| Australia | 0.771438 | 16 | 0.984375 | 7.153875 | 0.833709 | -0.062271 | Excellent | Excellent |
| New Zealand | 0.726179 | 15 | 0.937500 | 6.534121 | 0.740878 | -0.014699 | Excellent | Excellent |
| Japan | 0.684367 | 14 | 0.875000 | 6.150349 | 0.683394 | 0.000974 | Excellent | Excellent |
| Korea | 0.674748 | 13 | 0.812500 | 5.887147 | 0.643969 | 0.030779 | Good | Intermediate |
| Lebanon | 0.665460 | 12 | 0.750000 | 5.674490 | 0.612116 | 0.053344 | Good | Intermediate |
| Philippines | 0.625651 | 11 | 0.687500 | 5.488776 | 0.584299 | 0.041352 | Good | Intermediate |
| China | 0.596608 | 10 | 0.625000 | 5.318639 | 0.558814 | 0.037794 | Good | Intermediate |
| Saudi Arabia | 0.534678 | 9 | 0.562500 | 5.157311 | 0.534649 | 0.000028 | Good | Intermediate |
| India | 0.496772 | 8 | 0.500000 | 5.000000 | 0.511086 | -0.014314 | Moderate | Intermediate |
| Iraq | 0.453500 | 7 | 0.437500 | 4.842689 | 0.487523 | -0.034023 | Moderate | Intermediate |
| Jordan | 0.449017 | 6 | 0.375000 | 4.681361 | 0.463358 | -0.014341 | Moderate | Intermediate |
| Iran | 0.425119 | 5 | 0.312500 | 4.511224 | 0.437874 | -0.012754 | Moderate | Intermediate |
| Qatar | 0.418993 | 4 | 0.250000 | 4.325510 | 0.410056 | 0.008937 | Moderate | Intermediate |
| Chinese Taipei | 0.394020 | 3 | 0.187500 | 4.112853 | 0.378203 | 0.015817 | Moderate | Intermediate |
| Guam | 0.358924 | 2 | 0.125000 | 3.849651 | 0.338778 | 0.020145 | Poor | Poor |
| Syria | 0.224527 | 1 | 0.062500 | 3.465879 | 0.281294 | -0.056768 | Poor | Poor |

Note: q is the ascending WRSR rank used to form the cumulative proportion P. The terminal P value was corrected as 1 - 1/(4m) = 0.984375. The fitted model was WRSR = -0.2378 + 0.1498 × Probit (R² = 0.9524, F(1,14) = 280.32, P < 0.001). The three-level sensitivity scheme merges the Moderate and Good strata; it does not change WRSR values or team rankings.

**Supplementary Table S8. Cumulative-stage WRSR trajectories of the 16 teams**

| **Team** | **End of group stage** | **End of quarter-final phase** | **End of semifinals** | **Tournament end** |
| --- | --- | --- | --- | --- |
| Australia | 0.7884 (2) | 0.7418 (2) | 0.7585 (1) | 0.7714 (1) |
| China | 0.6342 (4) | 0.6428 (6) | 0.6129 (7) | 0.5966 (7) |
| Chinese Taipei | 0.4107 (12) | 0.3879 (14) | 0.3879 (14) | 0.3940 (14) |
| Guam | 0.3286 (15) | 0.3570 (15) | 0.3569 (15) | 0.3589 (15) |
| India | 0.4402 (11) | 0.4760 (10) | 0.4950 (9) | 0.4968 (9) |
| Iran | 0.5470 (9) | 0.5126 (9) | 0.4350 (12) | 0.4251 (12) |
| Iraq | 0.4053 (14) | 0.4411 (11) | 0.4566 (10) | 0.4535 (10) |
| Japan | 0.7477 (3) | 0.6698 (3) | 0.6819 (3) | 0.6844 (3) |
| Jordan | 0.4596 (10) | 0.4292 (12) | 0.4416 (11) | 0.4490 (11) |
| Korea | 0.5553 (8) | 0.6507 (5) | 0.6682 (4) | 0.6747 (4) |
| Lebanon | 0.5884 (5) | 0.6581 (4) | 0.6592 (5) | 0.6655 (5) |
| New Zealand | 0.8153 (1) | 0.7775 (1) | 0.7572 (2) | 0.7262 (2) |
| Philippines | 0.5674 (7) | 0.5916 (7) | 0.6187 (6) | 0.6257 (6) |
| Qatar | 0.4083 (13) | 0.4129 (13) | 0.4110 (13) | 0.4190 (13) |
| Saudi Arabia | 0.5799 (6) | 0.5193 (8) | 0.5339 (8) | 0.5347 (8) |
| Syria | 0.2236 (16) | 0.2320 (16) | 0.2255 (16) | 0.2245 (16) |

Note: Values are reported as WRSR (rank). Each snapshot uses all games available up to the stated tournament phase and retains all 16 teams as evaluation objects. The quarter-final phase includes qualification-to-quarter-finals and quarter-final games. Standardization ranges, entropy weights, indicator ranks, and WRSR values were re-estimated independently at each cumulative snapshot.

**Supplementary Table S9. Entropy weights re-estimated at each cumulative tournament snapshot**

| **Indicator** | **Direction** | **End of group stage** | **End of quarter-final phase** | **End of semifinals** | **Tournament end** |
| --- | --- | --- | --- | --- | --- |
| Points | Positive | 0.1048 | 0.0918 | 0.0935 | 0.0887 |
| 2P% | Positive | 0.0843 | 0.0703 | 0.0737 | 0.0734 |
| 3P% | Positive | 0.0572 | 0.0475 | 0.0477 | 0.0454 |
| FT% | Positive | 0.0951 | 0.1182 | 0.1132 | 0.1124 |
| Offensive rebounds | Positive | 0.1316 | 0.1237 | 0.1141 | 0.1141 |
| Defensive rebounds | Positive | 0.1069 | 0.0959 | 0.0893 | 0.0821 |
| Assists | Positive | 0.1216 | 0.1234 | 0.1242 | 0.1216 |
| Fouls | Negative | 0.0773 | 0.0926 | 0.0933 | 0.0928 |
| Turnovers | Negative | 0.0739 | 0.0650 | 0.0626 | 0.0611 |
| Steals | Positive | 0.0742 | 0.1046 | 0.1101 | 0.1191 |
| Blocks | Positive | 0.0731 | 0.0670 | 0.0782 | 0.0893 |

Note: Each column sums to 1.0000 subject to rounding. Re-estimation at each snapshot allows the indicator discrimination structure to update as additional tournament games become available.

**Supplementary Figure S2. Cumulative WRSR trajectories of China and Australia across tournament progression**

Note: Data labels show WRSR values with overall rank in parentheses (R). The quarter-final phase includes qualification-to-quarter-finals and quarter-finals. The trajectory is cumulative: each snapshot incorporates all games available up to that point, with the entropy-weighted WRSR model re-estimated across the same 16 teams.
